# Supplementary material for: Towards Universal Stimuli-Responsive Drug Delivery Systems: Pillar[5]arenes Synthesis and Self-Assembly into Nanocontainers with Tetrazole Polymers
Source: Nanomaterials (Basel). 2021 Apr 8;11(4):947. doi: 10.3390/nano11040947 (PMC8068209; doi:10.3390/nano11040947)

Supporting Information

for

Towards universal stimuli-responsive Drug Delivery Systems based on the tetrazole-containing polymers: synthesis of pillar[5]arenes and their self-assembly into nanocontainers

Dmitriy N. Shurpik, [1] Lyaysan I. Makhmutova, [1] Konstantin S. Usachev, [2] Daut R. Islamov, [3] Olga A. Mostovaya, [1] Anastasia A. Nazarova, [1] Valeriy N. Kizhnyaev [4] and Ivan I. Stoikov [1,]*

[1] Kazan Federal University, A.M. Butlerov Chemical Institute, 420008 Kremlevskaya, 18, Kazan, Russian Federation

[2] Kazan Federal University, Institute of Fundamental Medicine and Biology, 420008 Kremlevskaya, 18, Kazan, Russian Federation

[3] Russian Academy of Sciences, Arbuzov Institute of Organic and Physical Chemistry, FRC Kazan Scientific Center, 420088, Arbuzov st., 8, Kazan, Russian Federation

[4] Irkutsk State University, 664003 K. Marksa, 1, Irkutsk, Russian Federation

E-mail: Ivan.Stoikov@mail.ru

* Corresponding author. Tel.: +7-8432-337463; fax: +7-8432-752253; e-mail: Ivan.Stoikov@mail.ru

Table of contents

1. NMR, MALDI TOF MS, IR spectra of the compounds 6-12 3

2. Crystal data 20

3. Figure S24. UV spectra and Bindfit (Fit data to 1:1, 1:2 and 2:1 Host-Guest equilibria)...21

4. Table S3. Dynamic light scattering. 29

5. Scanning electron microscopy..34

6. Fluorescence spectra..39

1. NMR, MALDI TOF MS, IR spectra of the compounds 6-12

Figure S1. ^{1}H NMR spectrum of 4,8,14,18,23,26,28,31,32,35-deca-(4-methylbenzylsulfonate-1-ethoxy)-pillar[5]arene (**6**), $CDCl_3$, 298 K, 400 MHz.

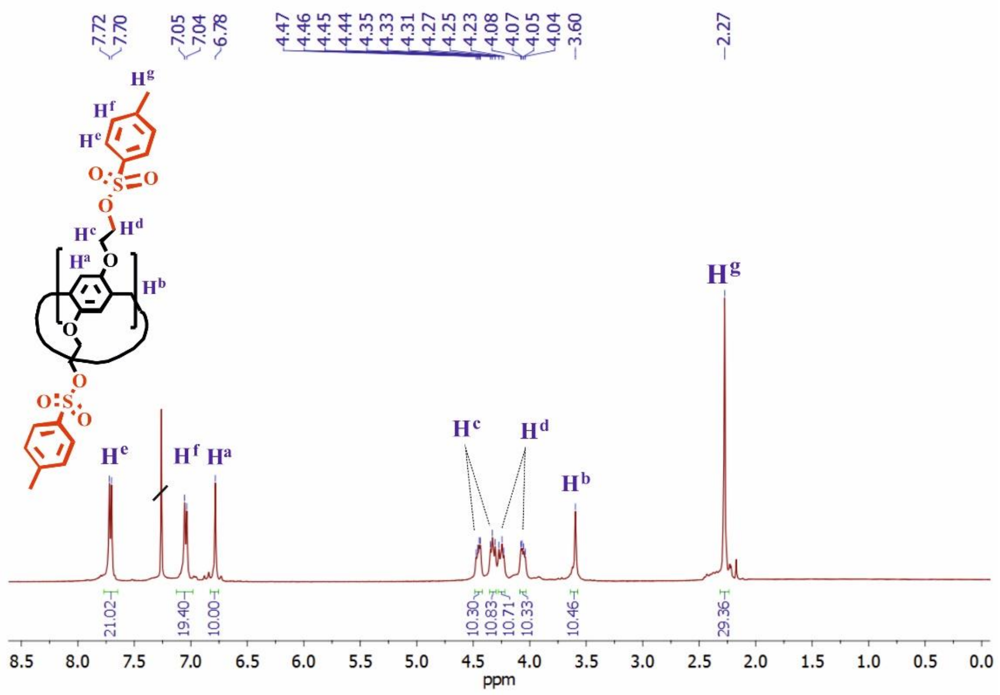

Figure S2. ^{13}C NMR spectrum of 4,8,14,18,23,26,28,31,32,35-deca-(4-methylbenzylsulfonate-1-ethoxy)-pillar[5]arene (**6**), $CDCl_3$, 298 K, 100 MHz.

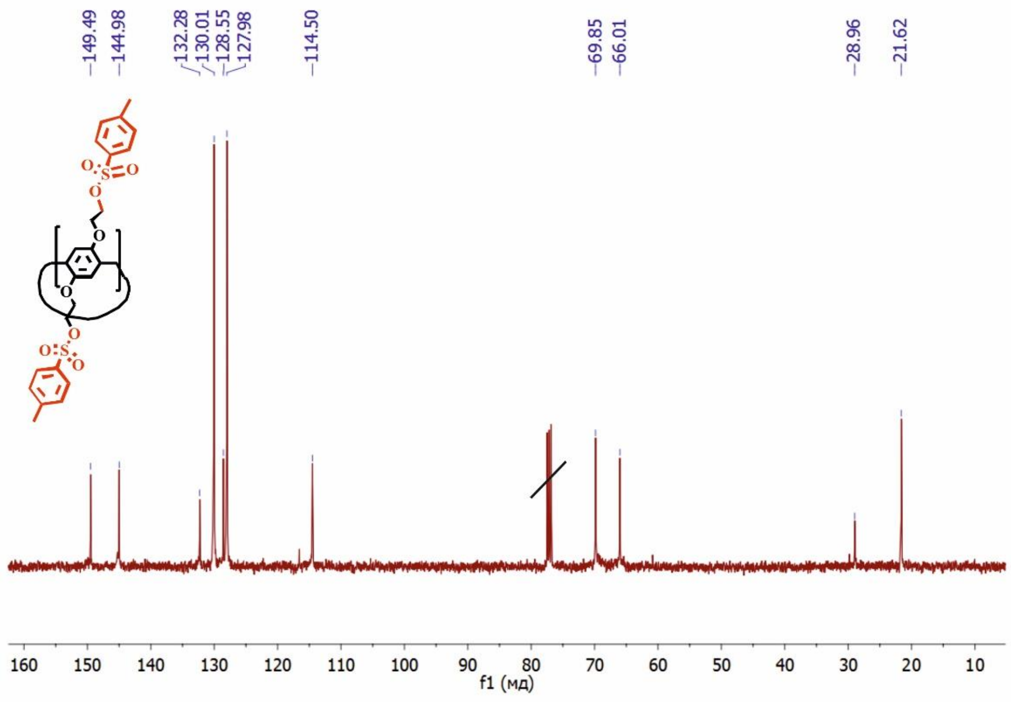

Figure S3. Mass spectrum (MALDI-TOF, 4-nitroaniline matrix) of 4,8,14,18,23,26,28,31,32,35-deca-(4-methylbenzylsulfonate-1-ethoxy)-pillar[5]arene (**6**).

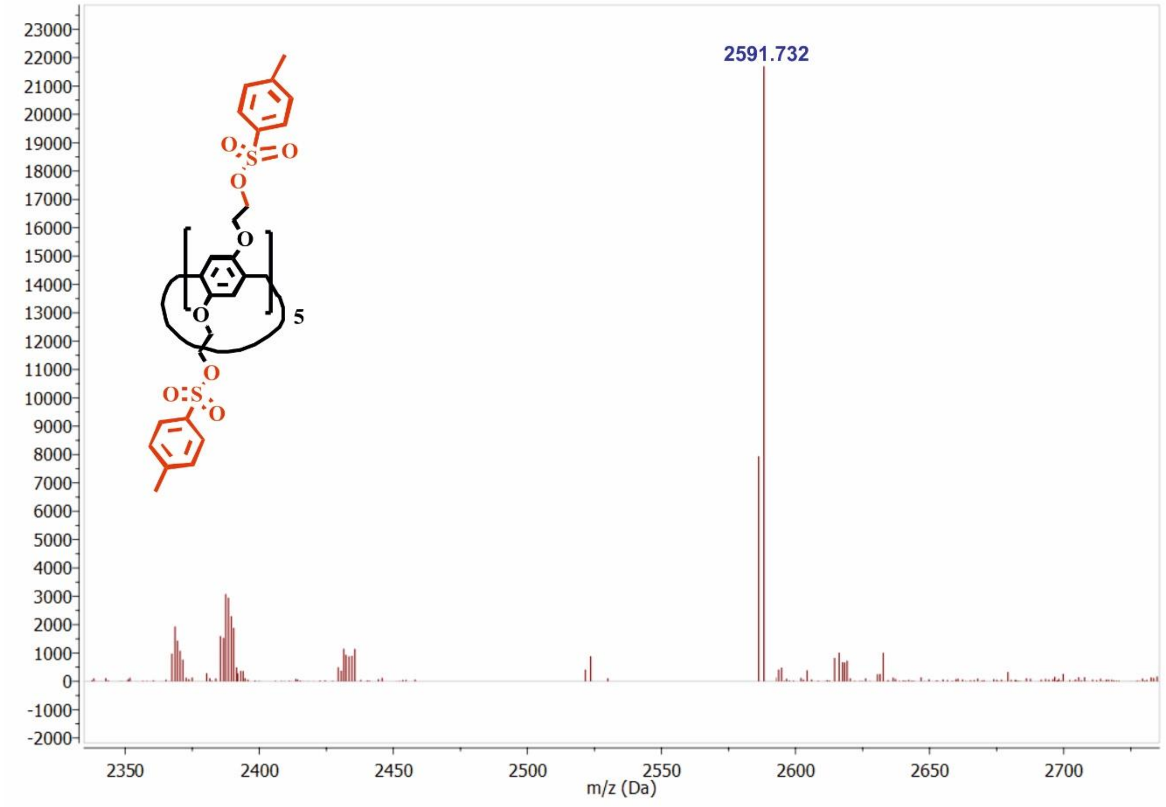

Figure S4. IR spectrum of 4,8,14,18,23,26,28,31,32,35-deca-(4-methylbenzylsulfonate-1-ethoxy)-pillar[5]arene (**6**).

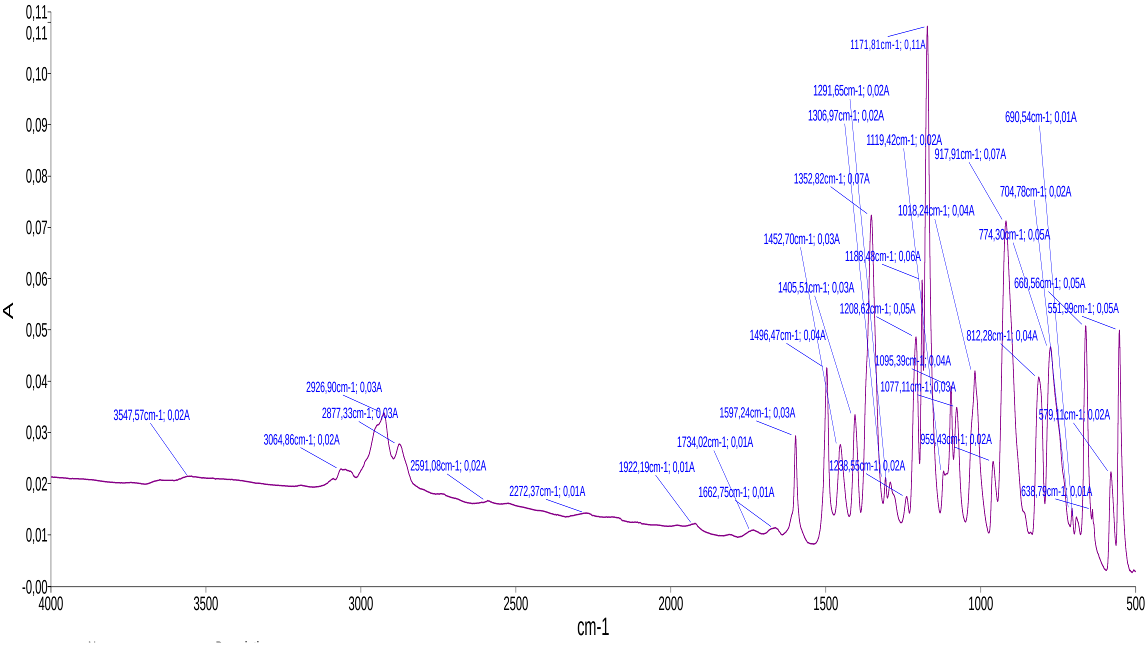

Figure S5. ^{1}H NMR spectrum of 4,8,14,18,23,26,28,31,32,35-deca-[(isoindoline-1,3-dione)propoxy]-pillar[5]arene (**11**), $CDCl_3$, 298 K, 400 MHz.

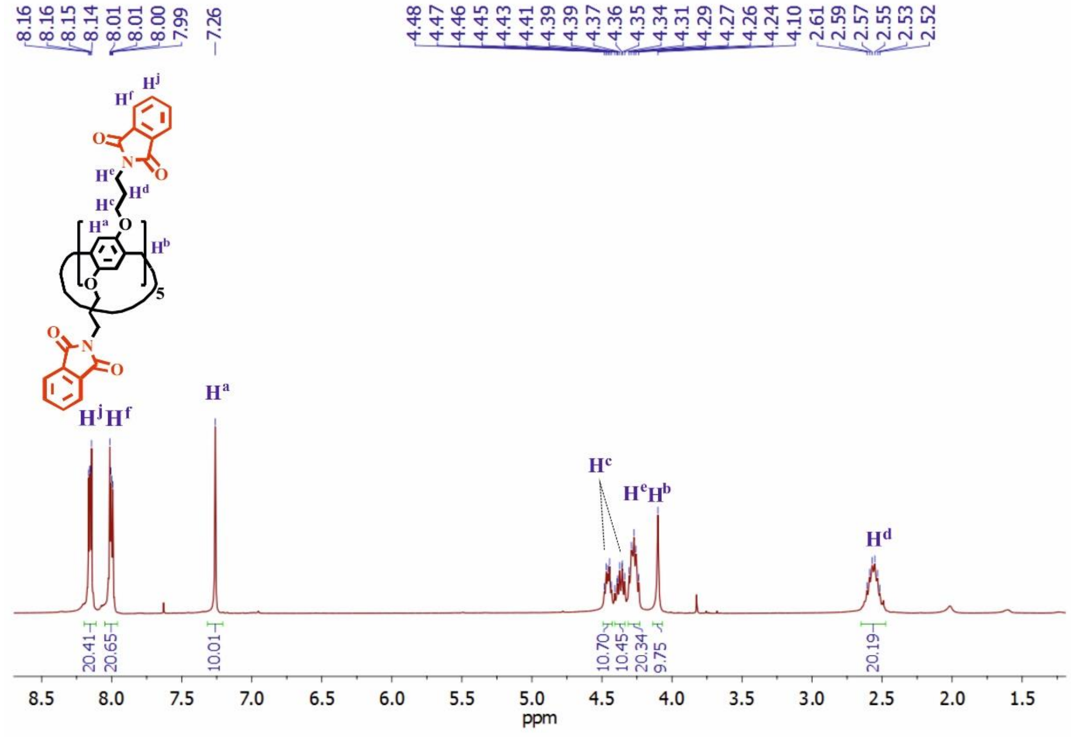

Figure S6. ^{13}C NMR spectrum of 4,8,14,18,23,26,28,31,32,35-deca-[(isoindoline-1,3-dione)propoxy]-pillar[5]arene (**11**), $CDCl_3$, 298 K, 100 MHz.

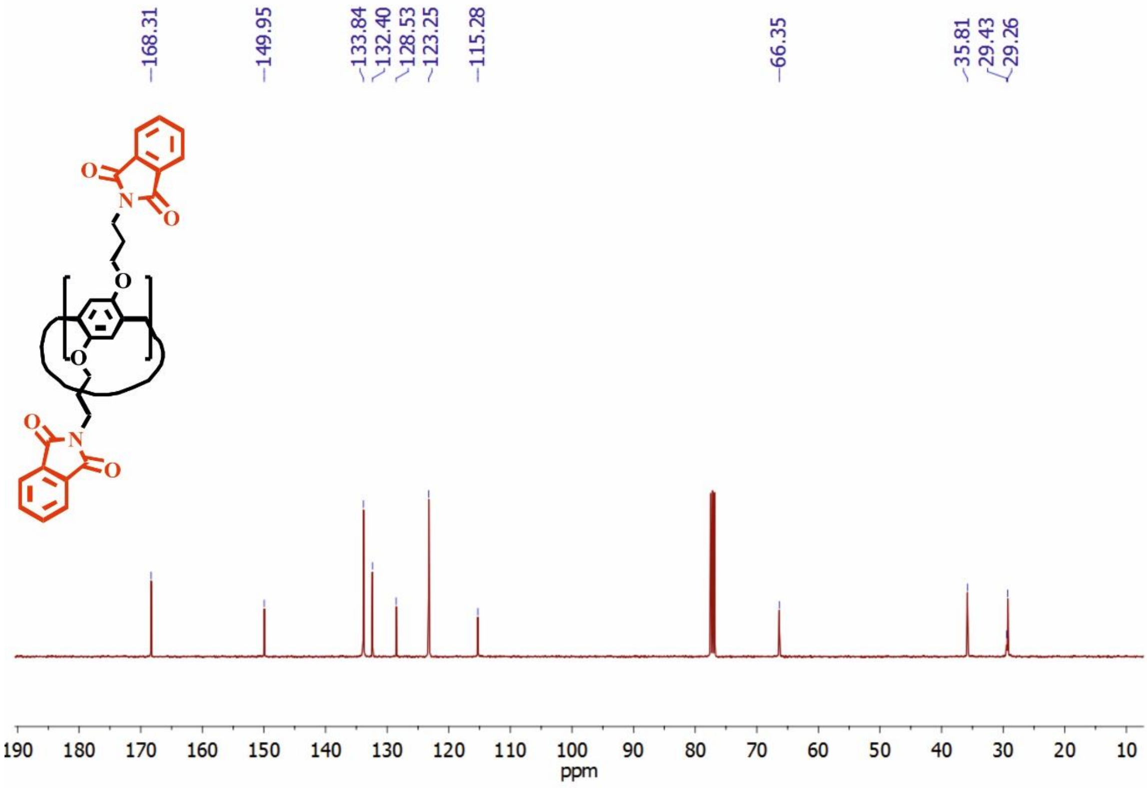

Figure S7. IR spectrum of 4,8,14,18,23,26,28,31,32,35-deca-[(isoindoline-1,3-dione)propoxy]-pillar[5]arene (**11**).

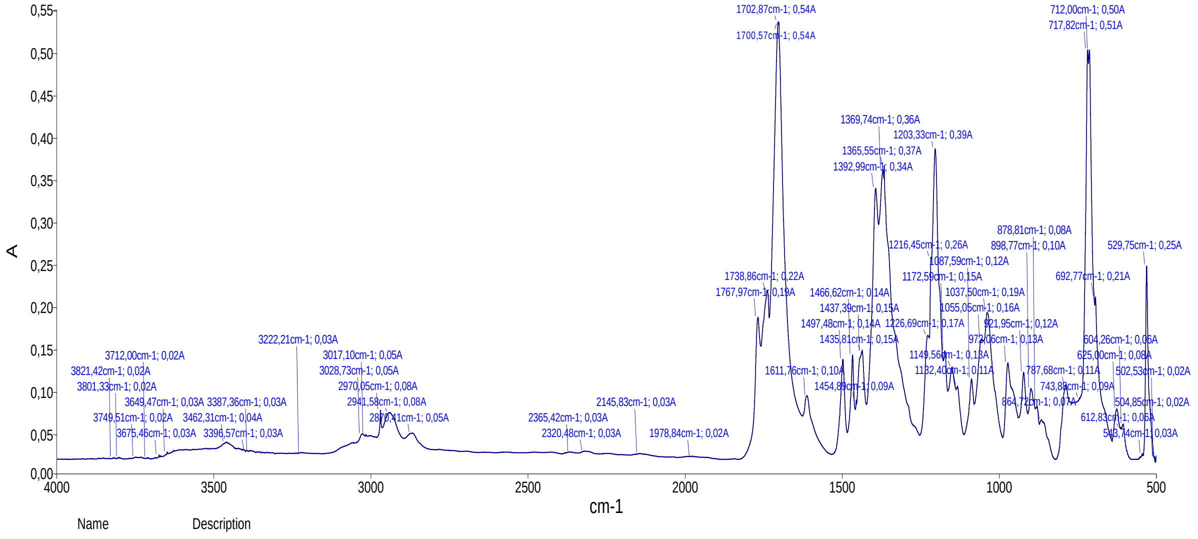

Figure S8. Mass spectrum (MALDI-TOF, 4-nitroaniline matrix) of 4,8,14,18,23,26,28,31,32,35-deca-[(isoindoline-1,3-dione)propoxy]-pillar[5]arene (**11**)

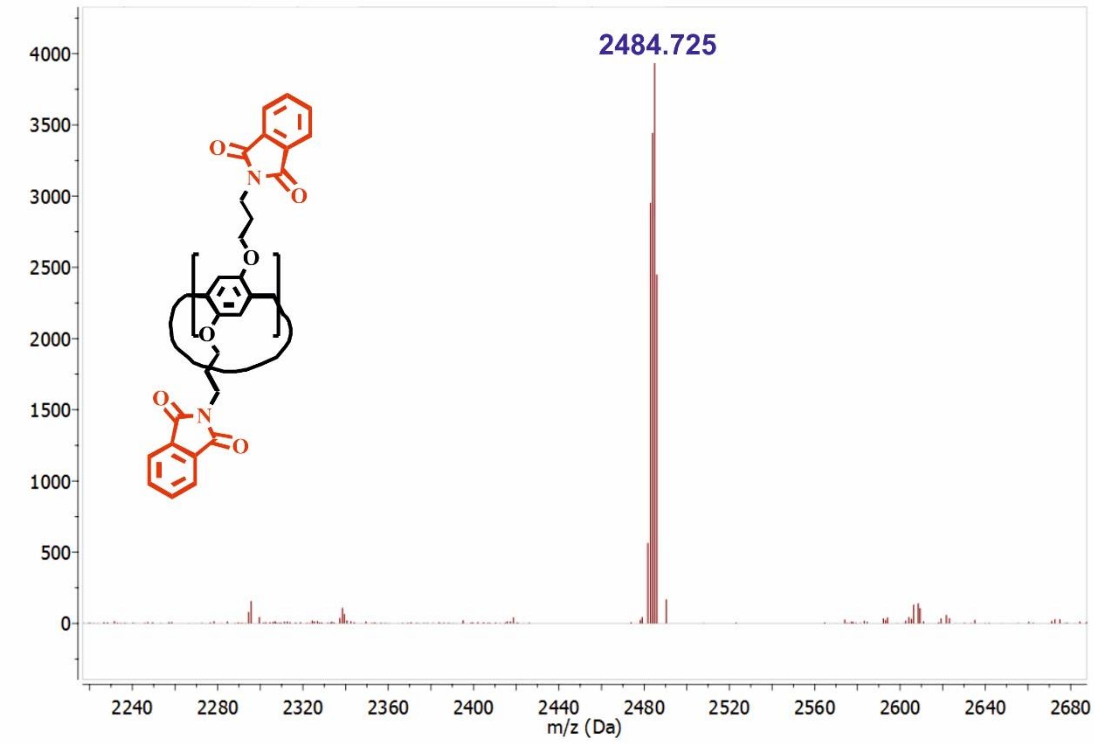

Figure S9. ^{1}H NMR spectrum of 4,8,14,18,23,26,28,31,32,35-deca-[2-(pyrrolidin-1-yl)ethoxy]-pillar[5]arene (7), $CDCl_3$, 298 K, 400 MHz.

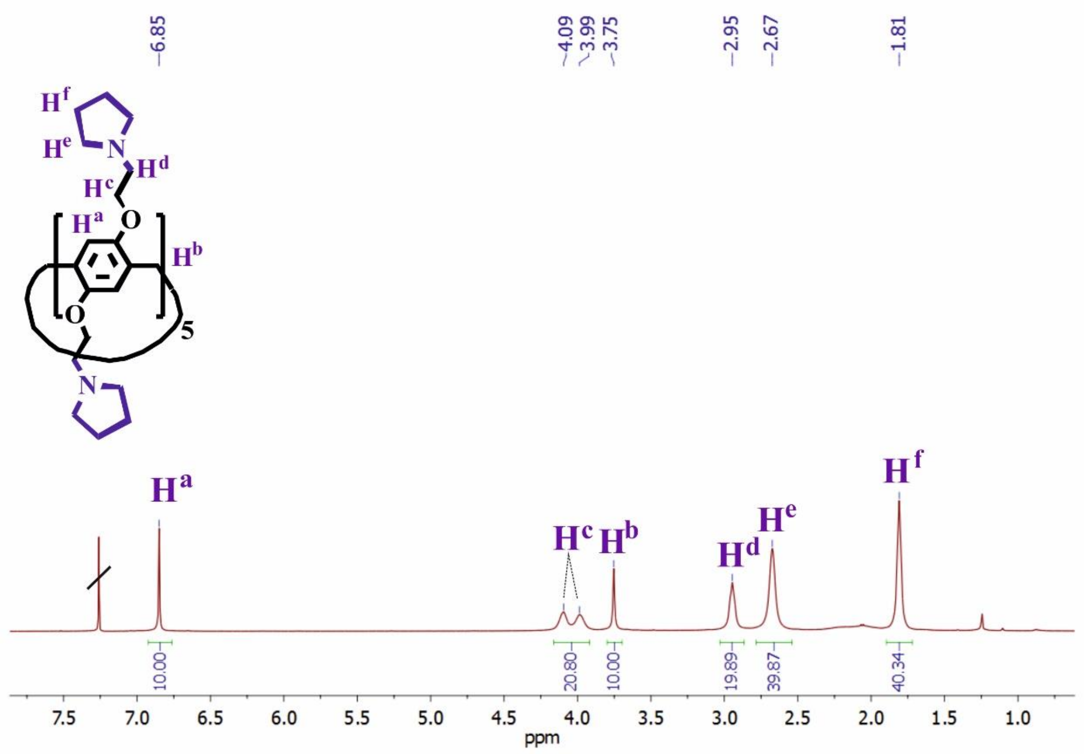

Figure S10. ^{13}C NMR spectrum of 4,8,14,18,23,26,28,31,32,35-deca-[2-(pyrrolidin-1-yl)ethoxy]-pillar[5]arene (**7**), $CDCl_3$, 298 K, 100 MHz.

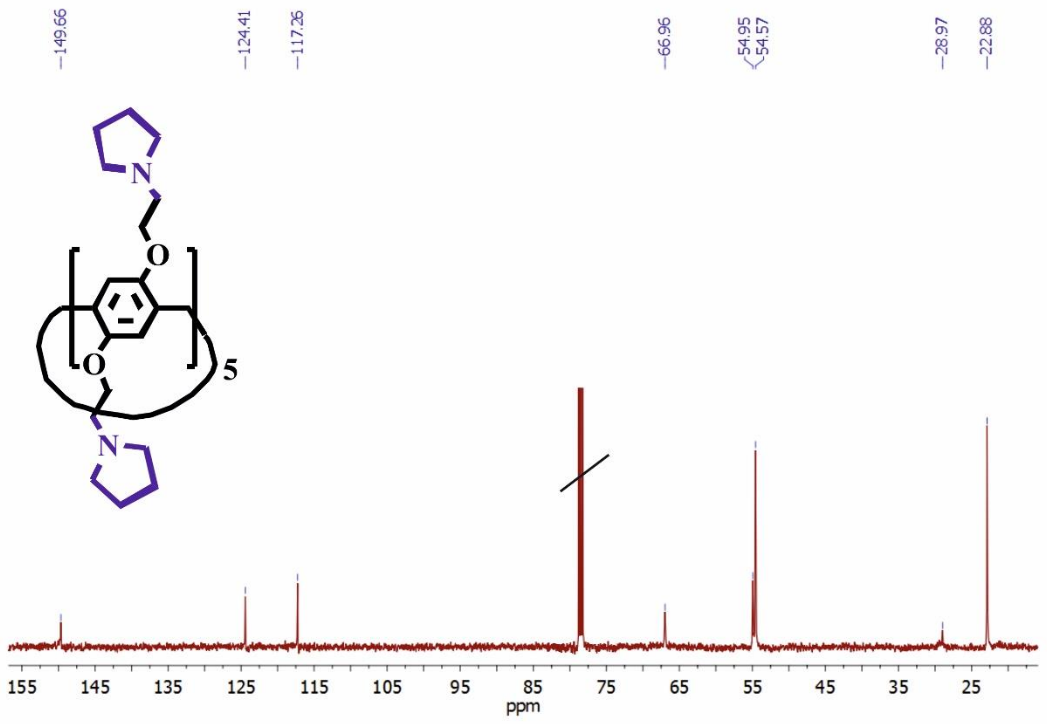

Figure S11. Mass spectrum (MALDI-TOF, 4-nitroaniline matrix) of 4,8,14,18,23,26,28,31,32,35-deca-[2-(pyrrolidin-1-yl)ethoxy]-pillar[5]arene (**7**).

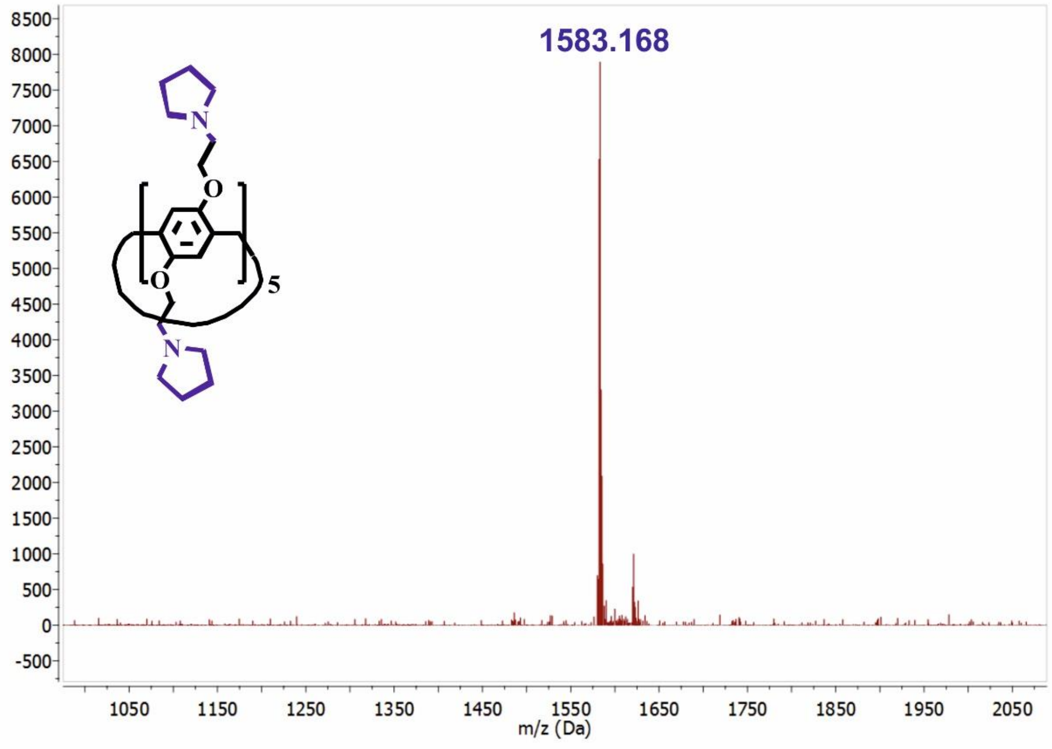

Figure S12. IR spectrum of 4,8,14,18,23,26,28,31,32,35-deca-[2-(pyrrolidin-1-yl)ethoxy]-pillar[5]arene (**7**).

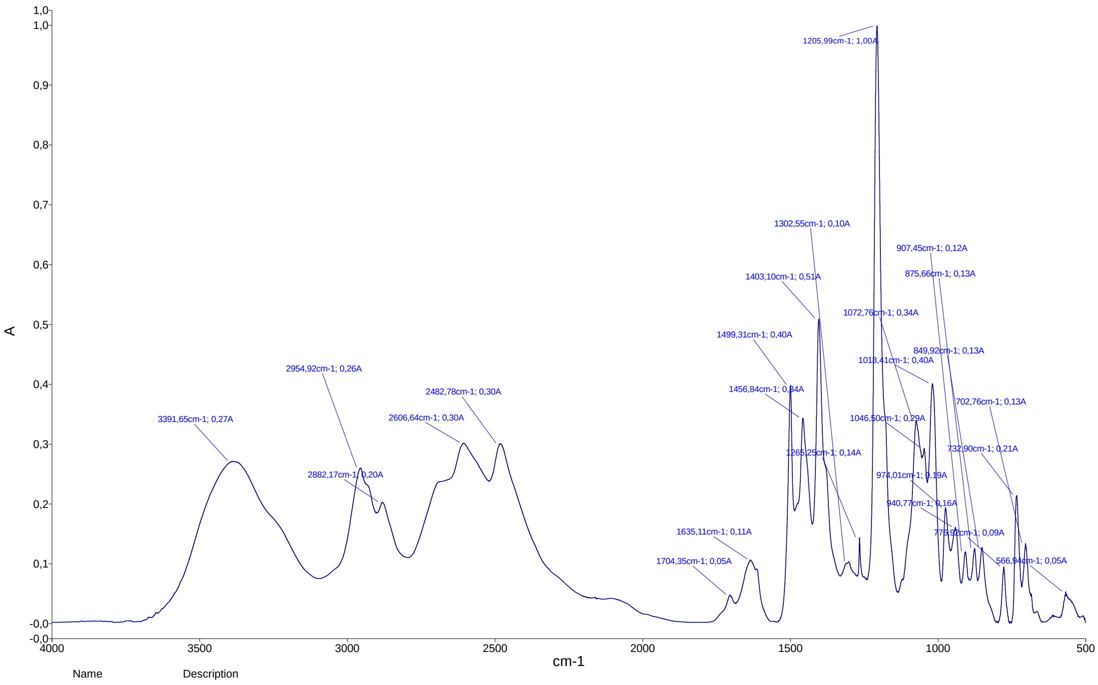

Figure S13. ^{1}H NMR spectrum of 4,8,14,18,23,26,28,31,32,35-deca-[2-(piperidin-1-yl)ethoxy]-pillar[5]arene (**8**) , $CDCl_3$, 298 K, 400 MHz.

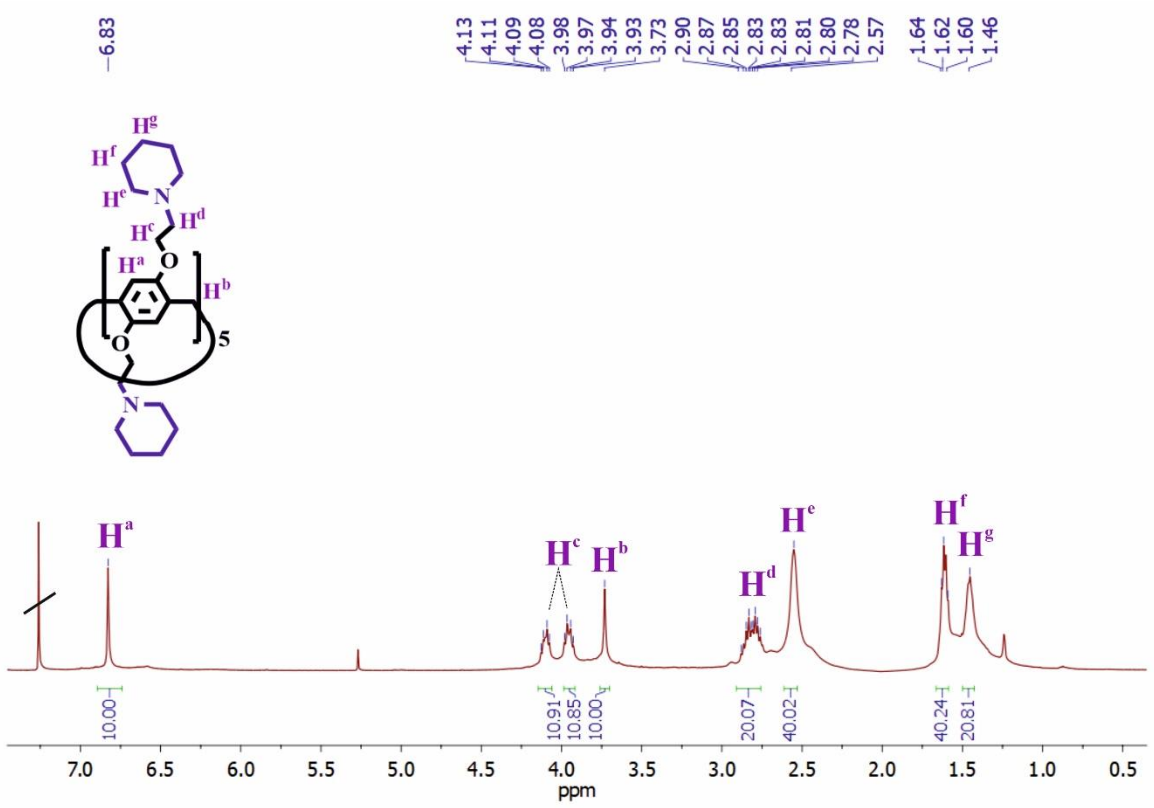

Figure S14. ^{13}C NMR spectrum of 4,8,14,18,23,26,28,31,32,35-deca-[2-(piperidin-1-yl)ethoxy]-pillar[5]arene (**8**), $CDCl_3$, 298 K, 100 MHz.

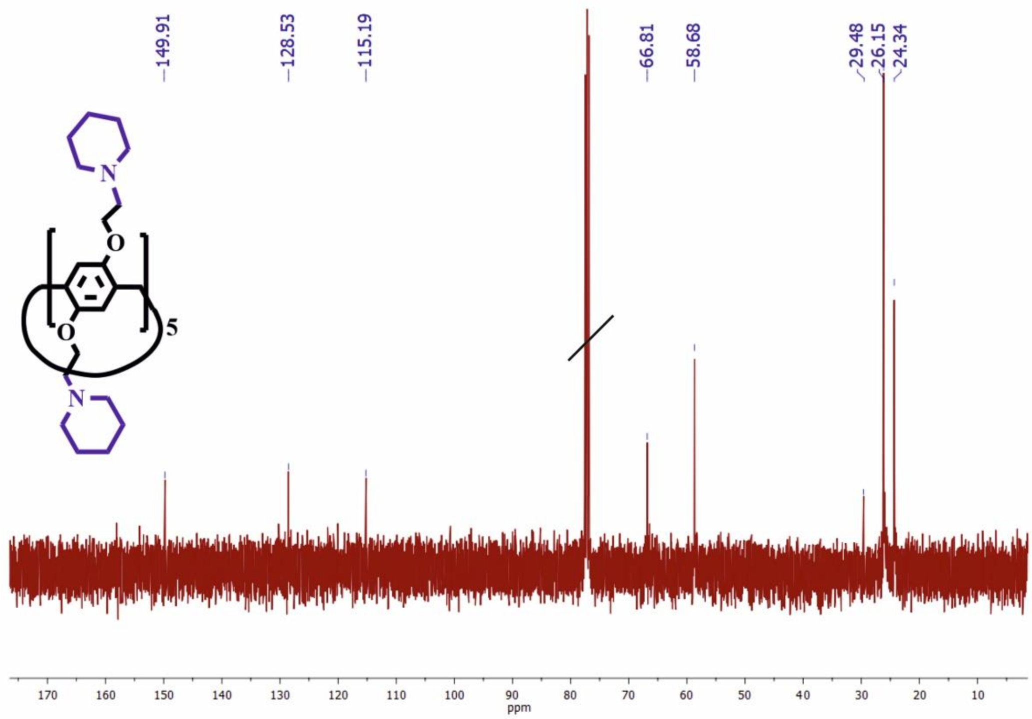

Figure S15. Mass spectrum (MALDI-TOF, 4-nitroaniline matrix) of 4,8,14,18,23,26,28,31,32,35-deca-[2-(piperidin-1-yl)ethoxy]-pillar[5]arene (**8**).

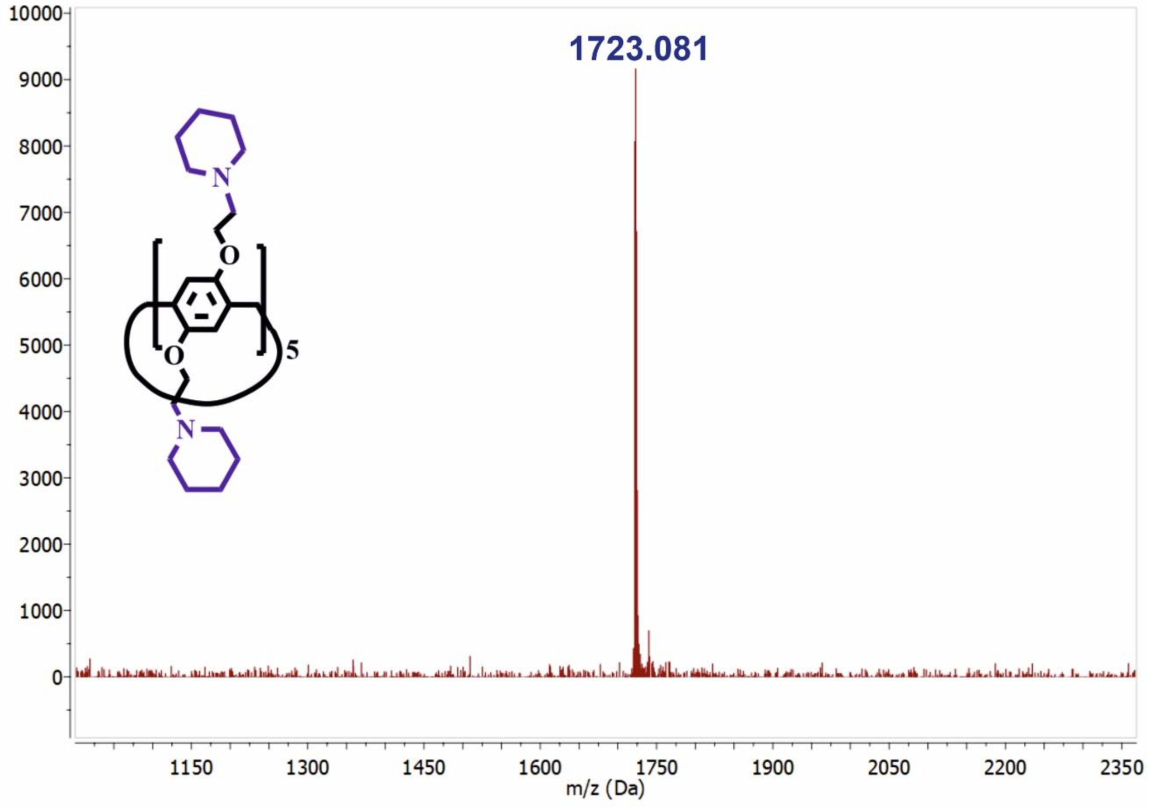

Figure S16. IR spectrum of 4,8,14,18,23,26,28,31,32,35-deca-[2-(piperidin-1-yl)ethoxy]-pillar[5]arene (**8**).

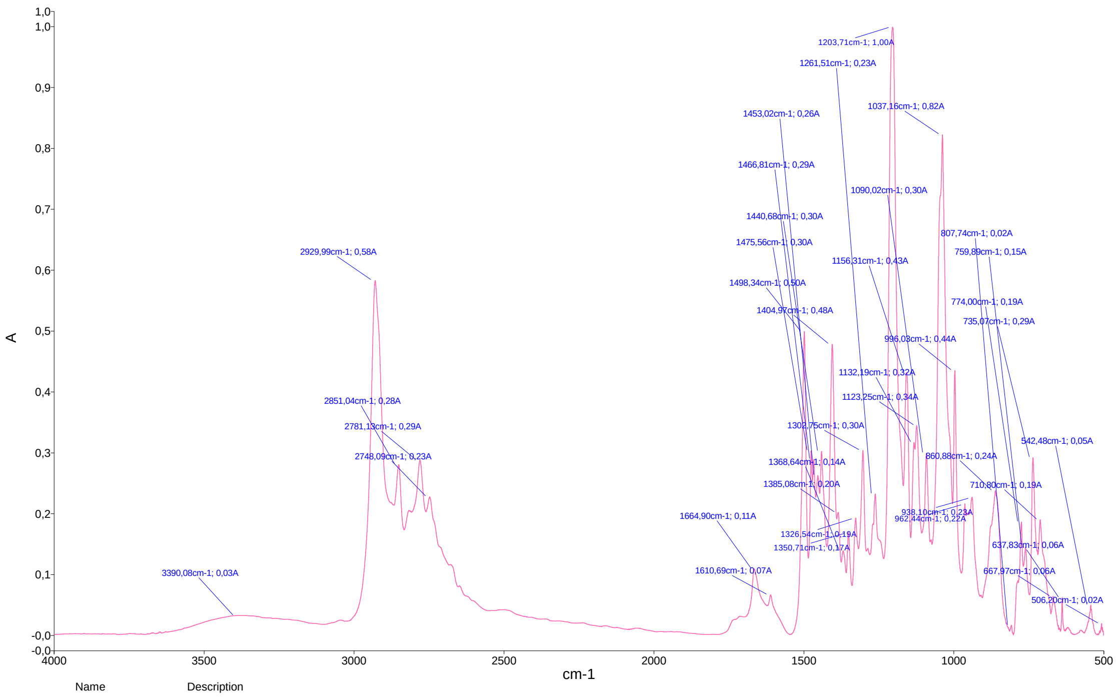

Figure S17. ^{1}H NMR spectrum of 4,8,14,18,23,26,28,31,32,35-deca-(2-morpholinoethoxy)-pillar[5]arene (**9**), $CDCl_3$, 298 K, 400 MHz.

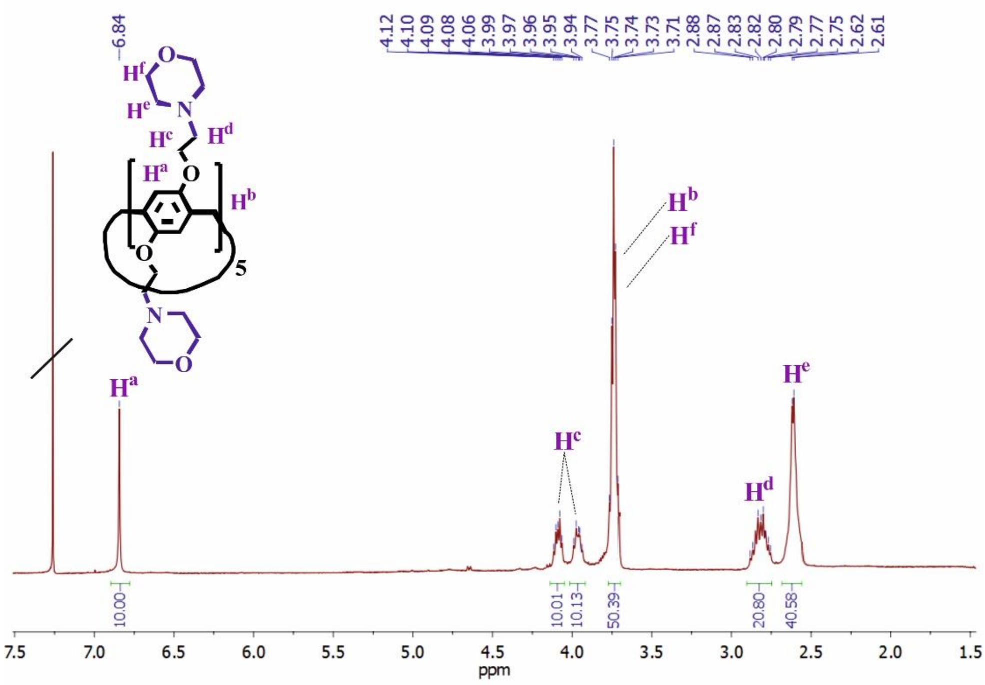

Figure S18. ^{13}C NMR spectrum of 4,8,14,18,23,26,28,31,32,35-deca-(2-morpholinoethoxy)-pillar[5]arene (**9**), $CDCl_3$, 298 K, 100 MHz.

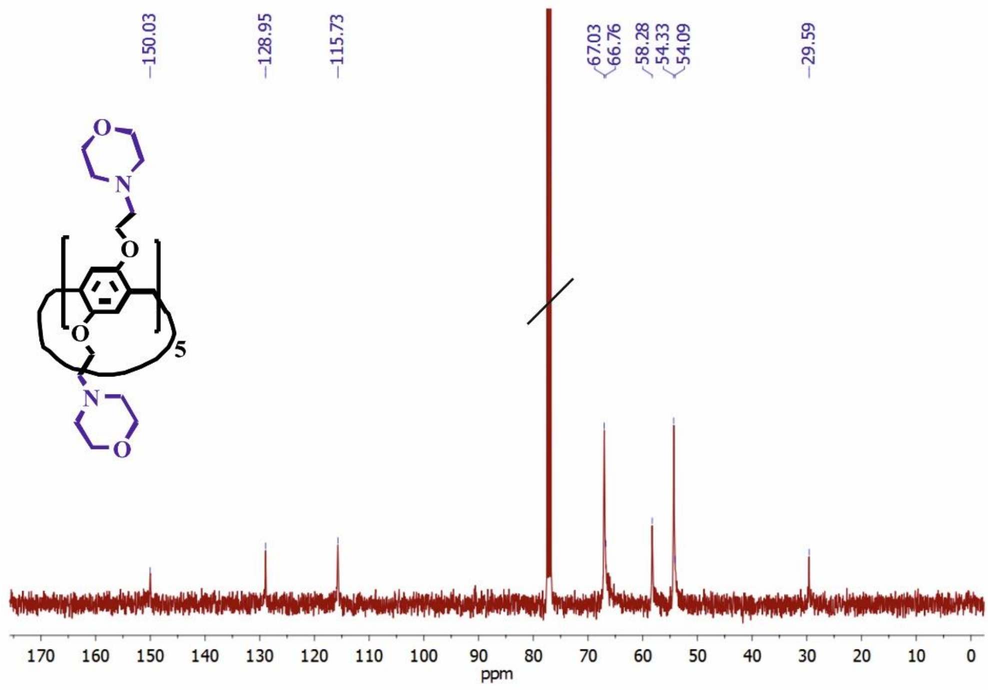

Figure S19. Mass spectrum (MALDI-TOF, 4-nitroaniline matrix) of 4,8,14,18,23,26,28,31,32,35-deca-(2-morpholinoethoxy)-pillar[5]arene (**9**).

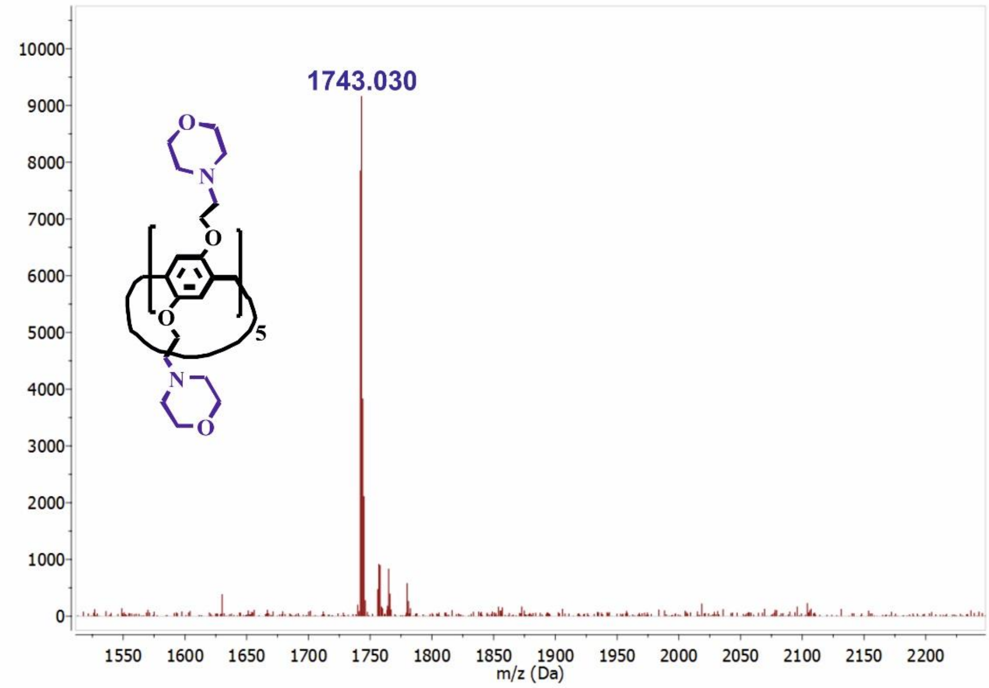

Figure S20. IR spectrum of 4,8,14,18,23,26,28,31,32,35-deca-(2-morpholinoethoxy)-pillar[5]arene (**9**).

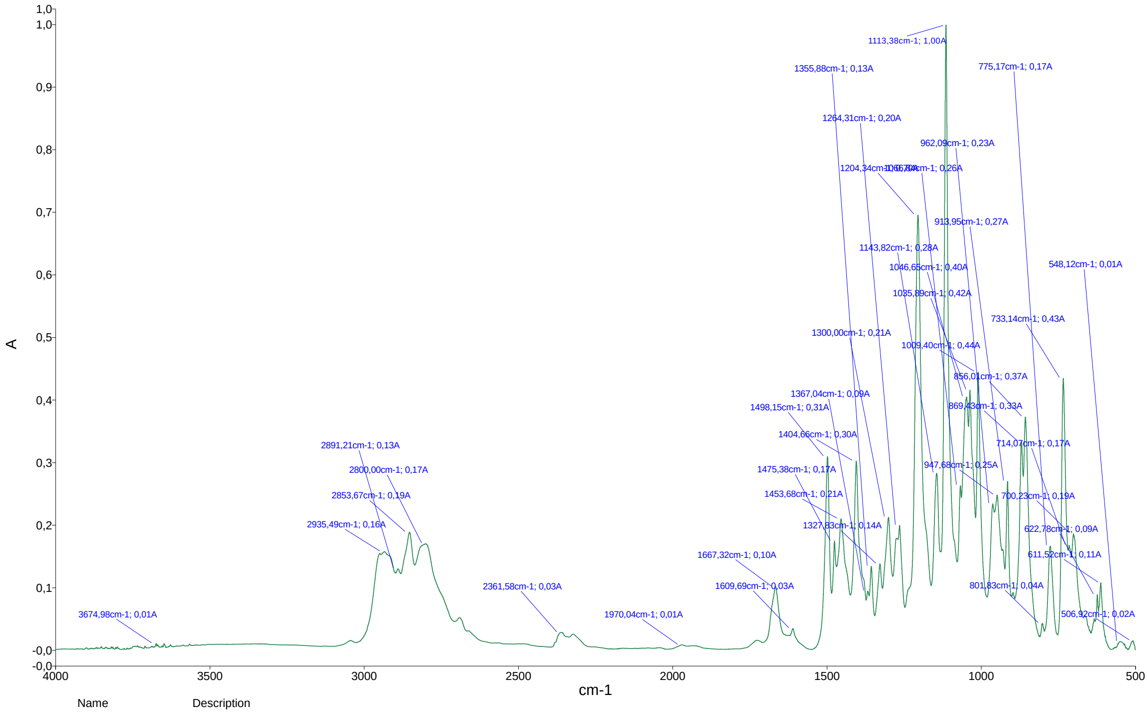

Figure S21. ^{1}H NMR spectrum of 4,8,14,18,23,26,28,31,32,35-deca-(aminopropyloxy)-pillar[5]arene (**12**), D_2O, 298 K, 400 MHz.

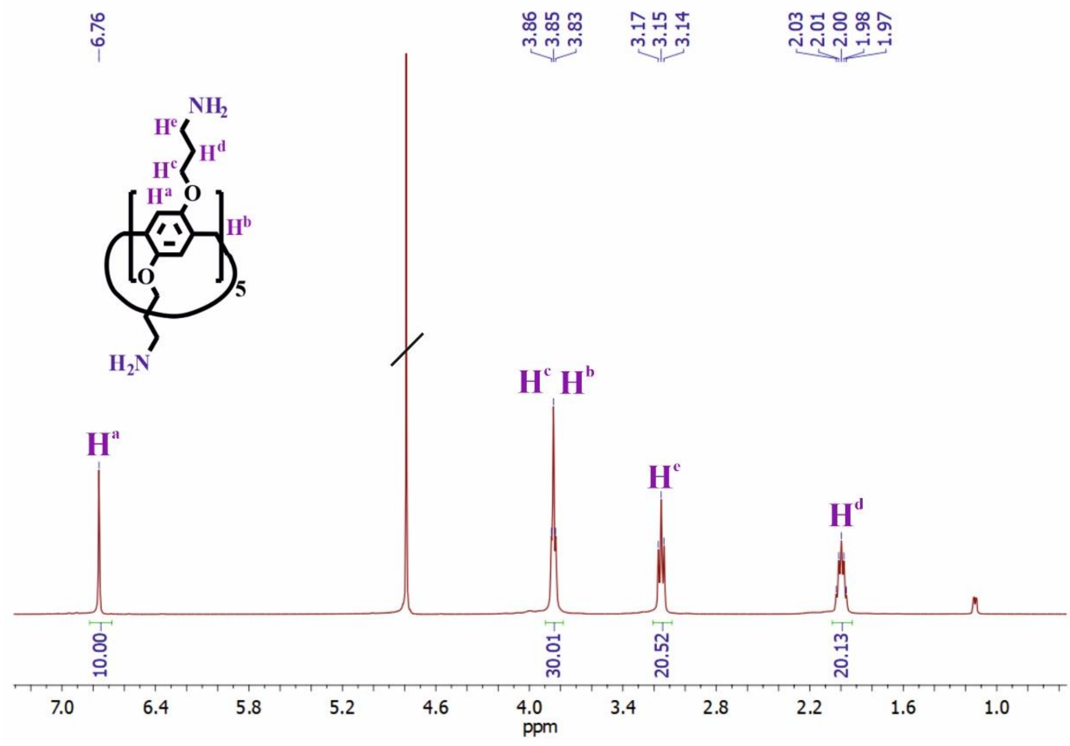

Figure S22.^{13}C NMR spectrum of 4,8,14,18,23,26,28,31,32,35-deca-(aminopropyloxy)-pillar[5]arene (12), D_2O, 298 K, 100 MHz.

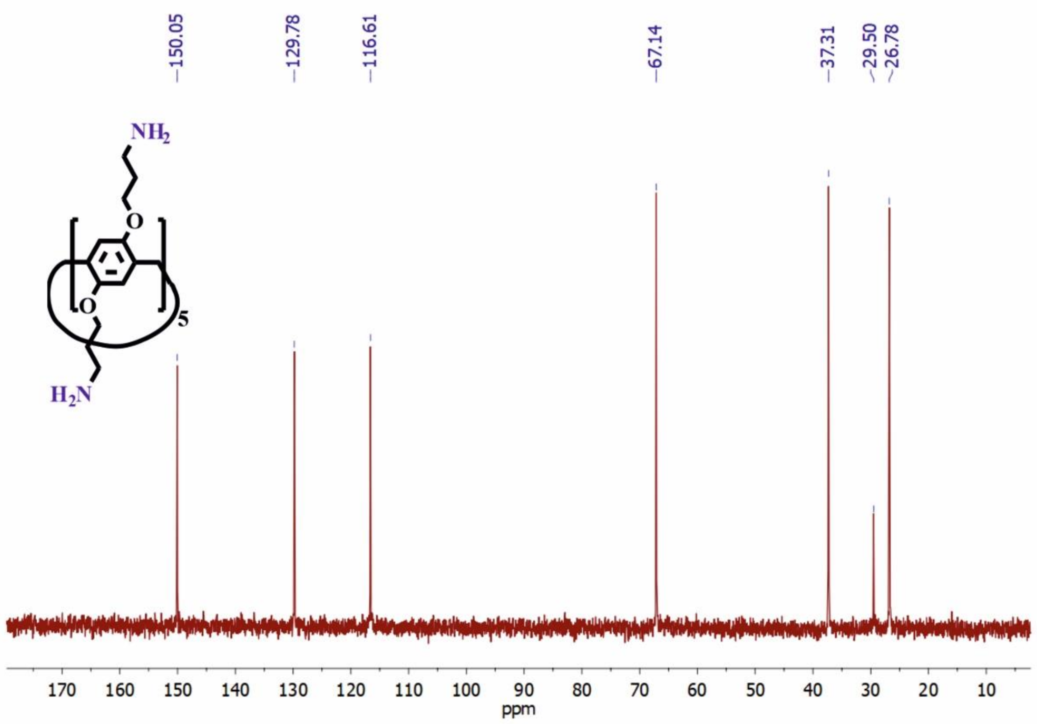

Figure S23. Mass spectrum (MALDI-TOF, 4-nitroaniline matrix) of 4,8,14,18,23,26,28,31,32,35-deca-(aminopropyloxy)-pillar[5]arene (**12**).

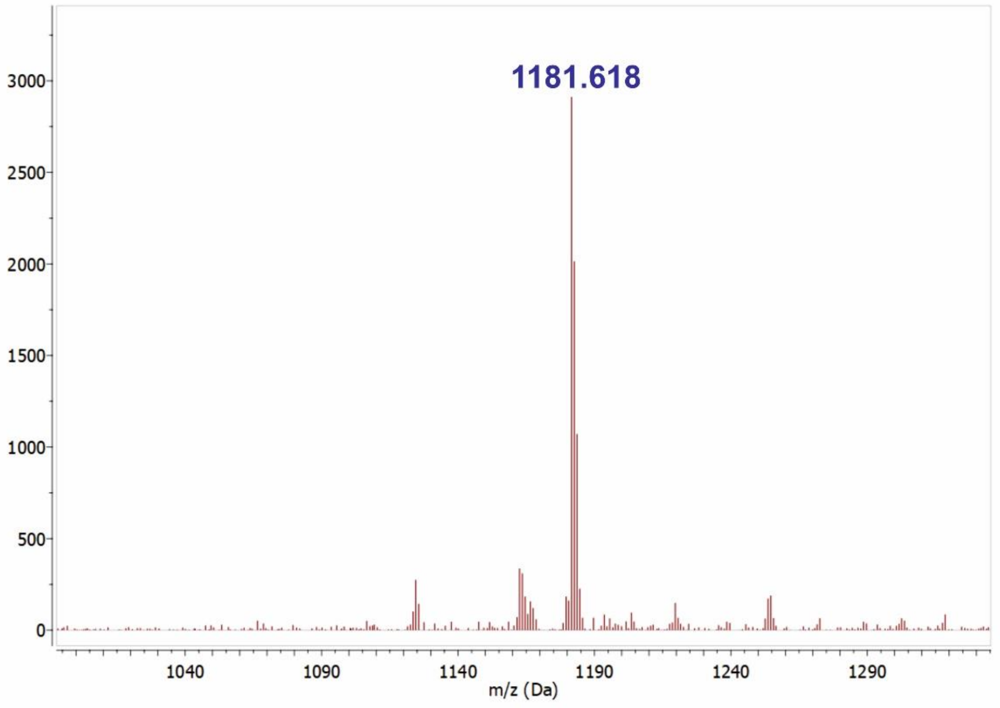

Figure S24. IR spectrum of 4,8,14,18,23,26,28,31,32,35-deca-(aminopropyloxy)-pillar[5]arene (**12**).

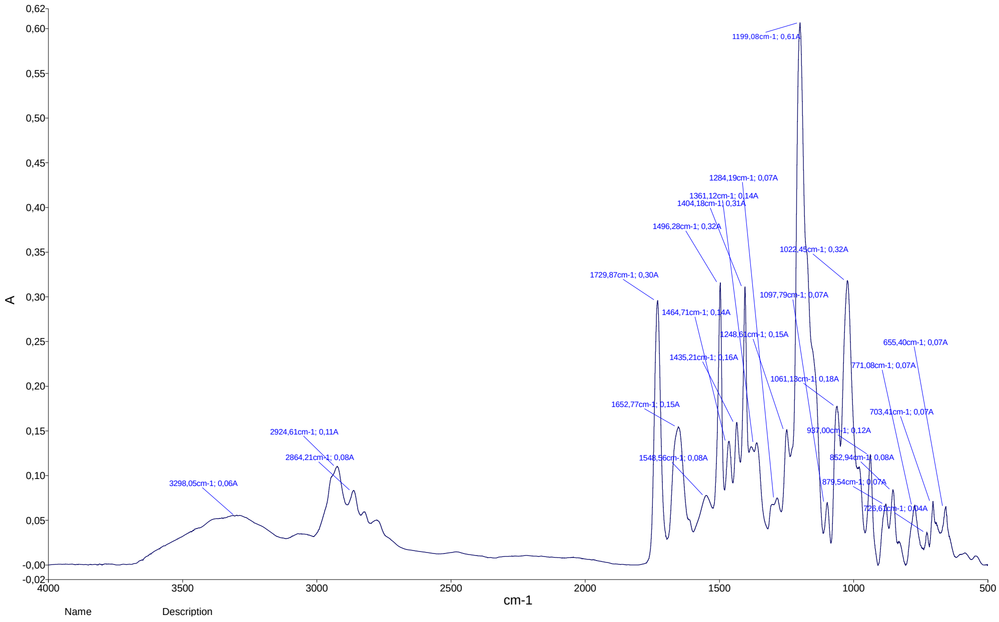

Figure S25. ^1H-^{13}C HSQC NMR spectrum of 4,8,14,18,23,26,28,31,32,35-deca-(4-methylbenzylsulfonate-1-ethoxy)-pillar[5]arene (6), $CDCl_3$, 298 K, 400 MHz.

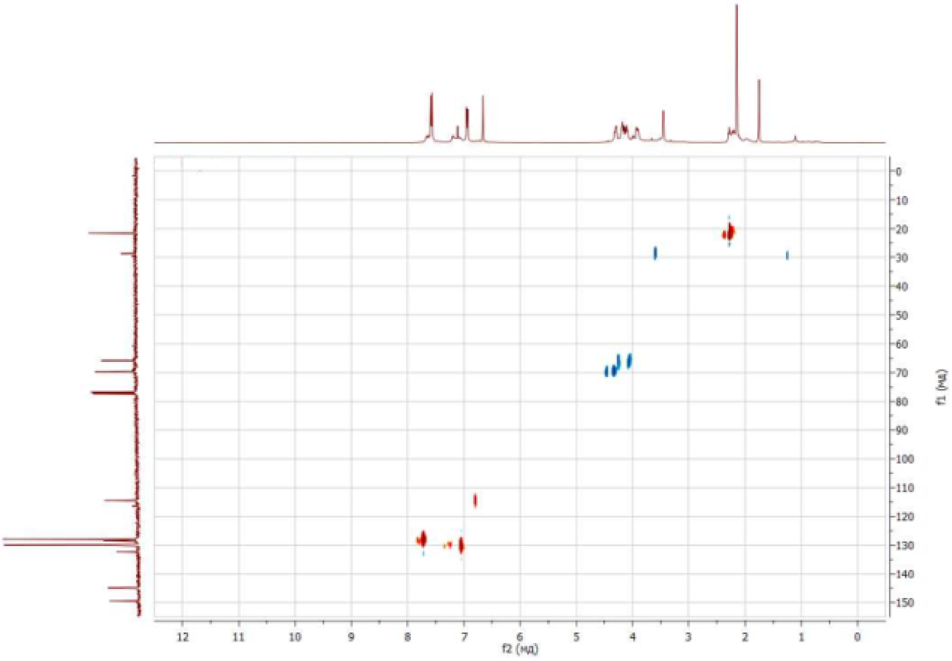

Table S1. Reaction conditions for the synthesis of target macrocycles **6** and **11** from starting compounds **5** and **10**, respectively.

Conditions	Compound s (equiv.)			Solvent	Temp. (°C)	Time (h)	Yield (%)
	Starting compound	Methylene Bridge reagent	Catalyst				
1	**5** (1 equiv.)	PFA (3 equiv.)	$BF_3 \times Et_2O$ (1 equiv.)	CH_2Cl-CH_2Cl	0-85	0.5-24	40-64
2	**5** (1 equiv.)	PFA (3 equiv.)	$BF_3 \times Et_2O$ (1 equiv.)	$CHCl_3$	0-50	2-24	5-20[1]
3	**5** (1 equiv.)	PFA (3 equiv.)	$BF_3 \times Et_2O$ (1 equiv.)	CH_2Cl_2	0-30	2-24	0
4	**5** (1 equiv.)	PFA (3 equiv.)	$AlBr_3$ (1 equiv.)	CH_2Cl-CH_2Cl	0-85	0.5-24	2-25
5	**5** (1 equiv.)	PFA (3 equiv.)	$AlBr_3$ (1 equiv.)	$CHCl_3$	0-50	2-24	4-40[1]
6	**5** (1 equiv.)	PFA (3 equiv.)	$AlBr_3$ (1 equiv.)	CH_2Cl_2	0-30	2-24	0
7	**5** (1 equiv.)	PFA (3 equiv.)	CF_3SO_3H (1 equiv.)	CH_2Cl-CH_2Cl	0-85	0.5-5	35-85
8	**5**	PFA	CF_3SO_3H	$CHCl_3$	0-50	0.5-5	7-45[1]

	(1 equiv.)	(3 equiv.)	(1 equiv.)				
9	**5** (1 equiv.)	PFA (3 equiv.)	CF_3SO_3H (1 equiv.)	CH_2Cl_2	0-30	0.5-5	0
10	**5** (1 equiv.)	PFA (3 equiv.)	CF_3COOH (1 equiv.)	CH_2Cl-CH_2Cl	0-85	0.5-5	1-10
11	**5** (1 equiv.)	PFA (3 equiv.)	CF_3COOH (1 equiv.)	$CHCl_3$	0-50	2-24	1-15[1]
12	**5** (1 equiv.)	PFA (3 equiv.)	CF_3COOH (1 equiv.)	CH_2Cl_2	0-30	2-24	0
13	**5** (1 equiv.)	Paraldehyde (3 equiv.)	$BF_3 \times Et_2O$ (1 equiv.)	CH_2Cl-CH_2Cl	0-85	0.5-24	5-16
14	**5** (1 equiv.)	Paraldehyde (3 equiv.)	CF_3SO_3H (1 equiv.)	CH_2Cl-CH_2Cl	0-85	0.5-24	10-20
15	**10** (1 equiv.)	PFA (3 equiv.)	$BF_3 \times Et_2O$ (1 equiv.)	CH_2Cl-CH_2Cl	0-85	0.5-24	21-53
16	**10** (1 equiv.)	PFA (3 equiv.)	$BF_3 \times Et_2O$ (1 equiv.)	$CHCl_3$	0-50	2-24	1-15[1]
17	**10** (1 equiv.)	PFA (3 equiv.)	$BF_3 \times Et_2O$ (1 equiv.)	CH_2Cl_2	0-30	2-24	0
18	**10** (1 equiv.)	PFA (3 equiv.)	$AlBr_3$ (1 equiv.)	CH_2Cl-CH_2Cl	0-85	0.5-24	1-17
19	**10** (1 equiv.)	PFA (3 equiv.)	$AlBr_3$ (1 equiv.)	$CHCl_3$	0-50	2-24	1-10[1]
20	**10** (1 equiv.)	PFA (3 equiv.)	$AlBr_3$ (1 equiv.)	CH_2Cl_2	0-30	2-24	0
21	**10** (1 equiv.)	PFA (3 equiv.)	CF_3SO_3H (1 equiv.)	CH_2Cl-CH_2Cl	0-85	0.5-5	15-74
22	**10** (1 equiv.)	PFA (3 equiv.)	CF_3SO_3H (1 equiv.)	$CHCl_3$	0-50	0.5-5	1-17[1]
23	**10** (1 equiv.)	PFA (3 equiv.)	CF_3SO_3H (1 equiv.)	CH_2Cl_2	0-30	0.5-5	0
24	**10** (1 equiv.)	PFA (3 equiv.)	CF_3COOH (1 equiv.)	CH_2Cl-CH_2Cl	0-85	0.5-5	0
25	**10** (1 equiv.)	PFA (3 equiv.)	CF_3COOH (1 equiv.)	$CHCl_3$	0-50	2-24	0
26	**10** (1 equiv.)	PFA (3 equiv.)	CF_3COOH (1 equiv.)	CH_2Cl_2	0-30	2-24	0
27	**10**	Paraldehyde	$BF_3 \times Et_2O$	CH_2Cl-CH_2Cl	0-85	0.5-24	5-10

	(1 equiv.)	(3 equiv.)	(1 equiv.)				
28	**10** (1 equiv.)	Paraldehyde (3 equiv.)	CF_3SO_3H (1 equiv.)	CH_2Cl-CH_2Cl	0-85	0.5-24	3-12

[1] According to data of NMR spectroscopy.

2. Crystal data

Table S2. Crystal data and structure refinement for **6**, **7** and **11**.

**Compound**	**6**	**7**	**11**
Formula	$C_{129}H_{136}N_2O_{40}S_{10}$	$C_{97}H_{143}N_{11}O_{10}$	$C_{150}H_{127}Cl_3N_{12}O_{30}$
Dcalc./ g cm^{-3}	1.392	1.167	1.082
μ/mm-1	2.315	0.596	1.056
Formula Weight	2674.99	1623.22	2683.98
Colour	clear brown	colourless	yellow
Shape	prism	plate	plate
Size/mm^3	0.27×0.18×0.11	0.15×0.09×0.02	0.42×0.23×0.15
T/*K*	99.9(2)	100.00(10)	99.99(10)
Crystal System	monoclinic	triclinic	triclinic
Space Group	*C*2/*c*	*P*-1	*P*-1
a/*Å*	27.2340(4)	12.4634(4)	19.9245(3)
b/*Å*	17.5786(3)	20.2507(12)	20.8844(3)
c/*Å*	26.8160(5)	20.7444(10)	21.69742(17)
α/°	90	63.291(6)	104.8530(11)
β/°	95.9945(14)	81.416(4)	98.5060(10)
γ/°	90	88.833(4)	104.3794(13)
V/Å^3	12767.6(4)	4618.2(4)	8234.8(2)
Z	4	2	2
Z'	0.5	1	1
Wavelength/ Å	1.54184	1.54184	1.54184
Radiation type	Cu K *α*	Cu K *α*	Cu K *α*
θ_{min}/°	2.997	2.414	2.163
θ_{max}/°	77.501	77.237	71.992
Measured Refl.	66822	63746	265502
Independent Refl.	13185	18772	32032
Reflections with *I* > 2(*I*)	11003	9154	22857
R_{int}	0.0400	0.1296	0.1000
Parameters	822	1093	1758
Restraints	0	0	63

Largest Peak	0.664	1.041	1.004
Deepest Hole	-0.431	-0.397	-1.328
GooF	1.075	1.023	1.436
wR_2 (all data)	0.2288	0.2831	0.3620
wR_2	0.2181	0.2235	0.3401
R_1 (all data)	0.0778	0.1672	0.1259
R_1	0.0706	0.0875	0.1096
CCDC Refcode	2027115	2027117	2027116

3. Figure S26. UV spectra and Bindfit (Fit data to 1:1, 1:2 and 2:1 Host-Guest equilibria)

UV-vis spectra of pillar[5]arene 7 (1×10^{-5} M) at different concentrations of PVTE.

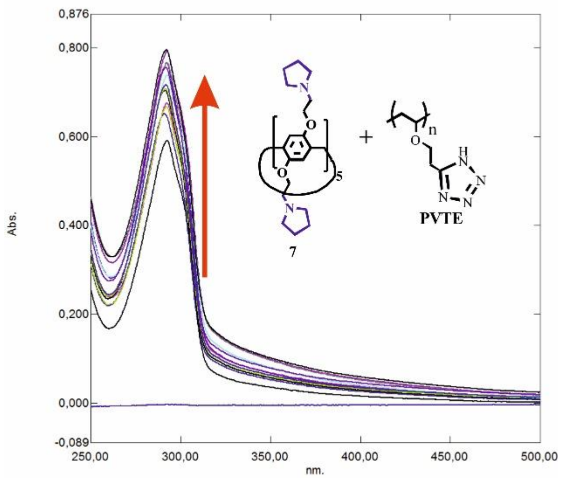

UV-vis spectra of pillar[5]arene 9 (1×10^{-5} M) at different concentrations of PVTE.

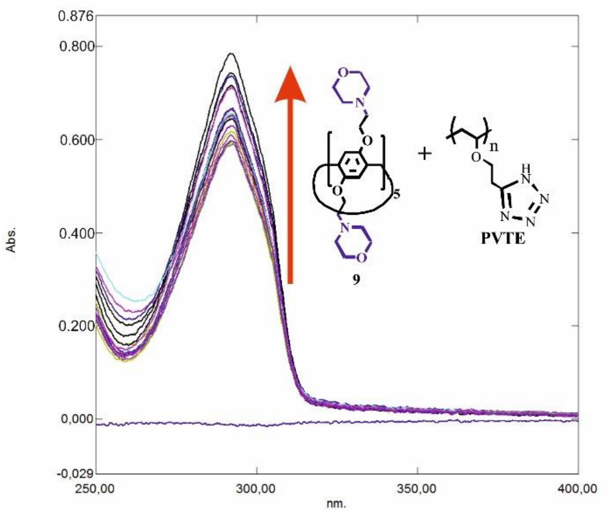

UV-vis spectra of Fluorescein (1×10^{-5} M) at different concentrations of pillar[5]arene 7.

UV-vis spectra of Fluorescein (1×10^{-5} M) at different concentrations of pillar[5]arene 9.

Fluorescence spectra of Fluorescein (1×10^{-5}M) at different concentrations of pillar[5]arene 7.

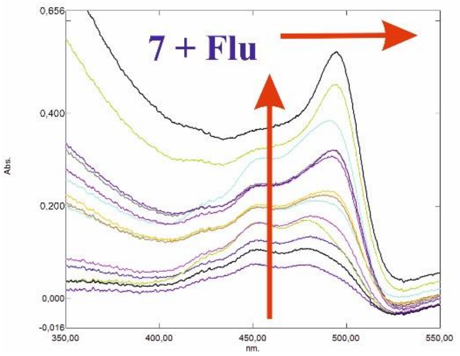
7 + Flu
Abs.
0,656
0,400
0,200
0,000
-0,016
350,00
400,00
450,00
500,00
550,00
nm.

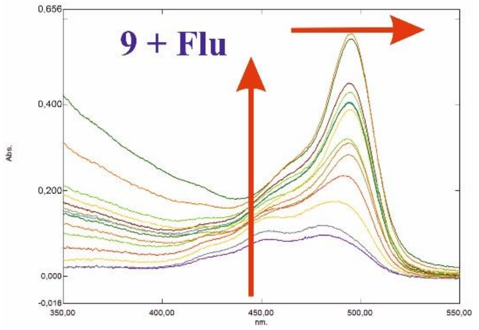
9 + Flu
Abs.
0,656
0,400
0,200
0,000
-0,016
350,00
400,00
450,00
500,00
550,00
nm.

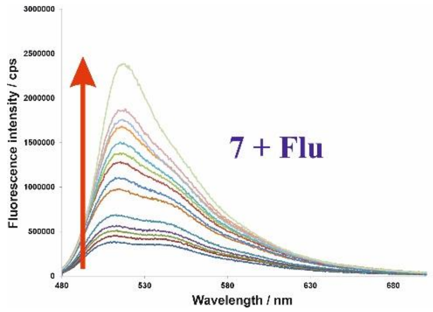
7 + Flu
Fluorescence intensity / cps
3000000
2500000
2000000
1500000
1000000
500000
0
480
530
580
630
680
Wavelength / nm

Figure S27. Screenshots taken from the summary window of the website supramolecular.org. This screenshots shows the raw data for UV-vis titration of 7 with PVTE, the data fitted to 1:1 binding model (A), 1:2 binding model (B) and 2:1 binding model (C).

(A)

Fitter: UV 1:1 | Fit | Summary | Save

Details

**Time to fit**	0.1862 s
**SSR**	1.7352e-4
**Fitted datapoints**	8
**Fitted params**	2

Parameters

Parameter (bounds)	Optimised	Error	Initial
K (0 → ∞)	2993.95 M^{-1}	± 9.1691 %	100.00 M^{-1}

Back Next

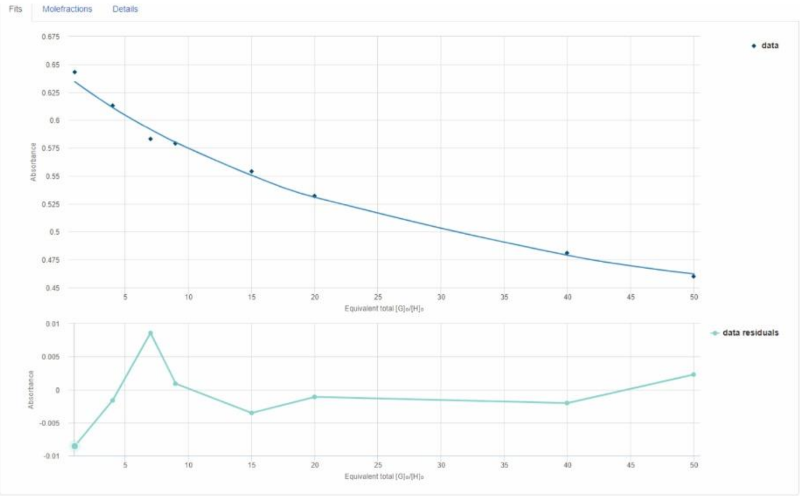

(B)

Fitter: UV 1:2 | Fit | Summary | Save

Details

**Time to fit**	0.6726 s
**SSR**	1.5484e-4
**Fitted datapoints**	8
**Fitted params**	4

Parameters

Parameter (bounds)	Optimised	Error	Initial
K_{11} (0 → ∞)	2970.97 M^{-1}	± 34.6617 %	1000.00 M^{-1}
K_{12} (0 → ∞)	-1413.44 M^{-1}	± -230.7551 %	100.00 M^{-1}

Back Next

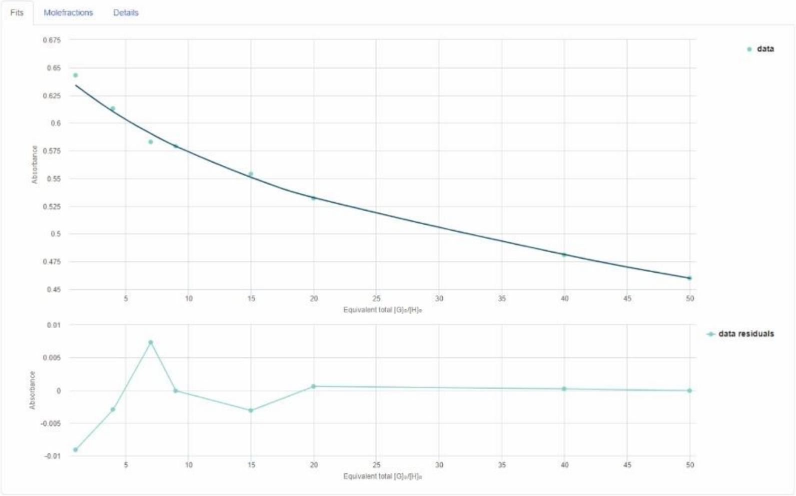

(C)

Fitter: UV 2:1 | Fit | Summary | Save

Details

**Time to fit**	0.9539 s
**SSR**	1.6112e-4
**Fitted datapoints**	8
**Fitted params**	4

Parameters

Parameter (bounds)	Optimised	Error	Initial
K_{11} (0 → ∞)	37.86 M^{-1}	± 80.3677 %	1000.00 M^{-1}
K_{21} (0 → ∞)	3241691.50 M^{-1}	± 124.8671 %	100.00 M^{-1}

Back Next

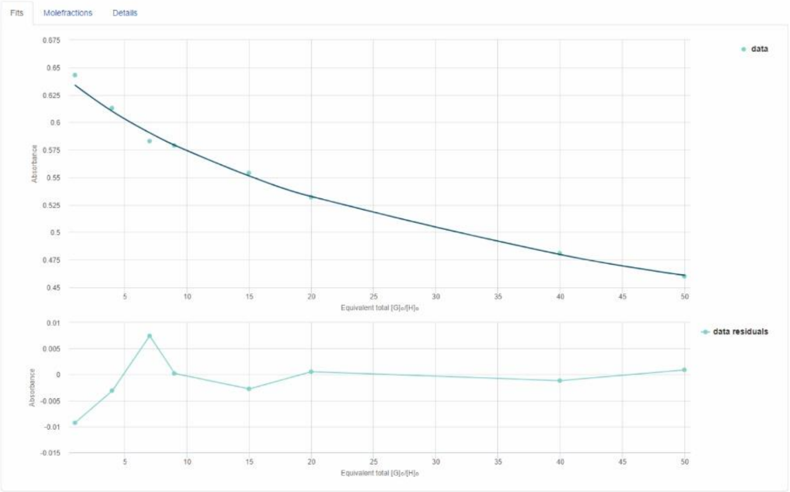

Figure S28. Screenshots taken from the summary window of the website supramolecular.org. This screenshots shows the raw data for UV-vis titration of 9 with PVTE, the data fitted to 1:1 binding model (A), 1:2 binding model (B) and 2:1 binding model (C).

(A)

Fitter: UV 1:1 | Fit | Summary | Save

Details

**Time to fit**	0.1815 s
**SSR**	1.8832e-6
**Fitted datapoints**	6
**Fitted params**	2

Parameters

Parameter (bounds)	Optimised	Error	Initial
K (0 → ∞)	1898.15 M^{-1}	± 10.7670 %	100.00 M^{-1}

Back | Next

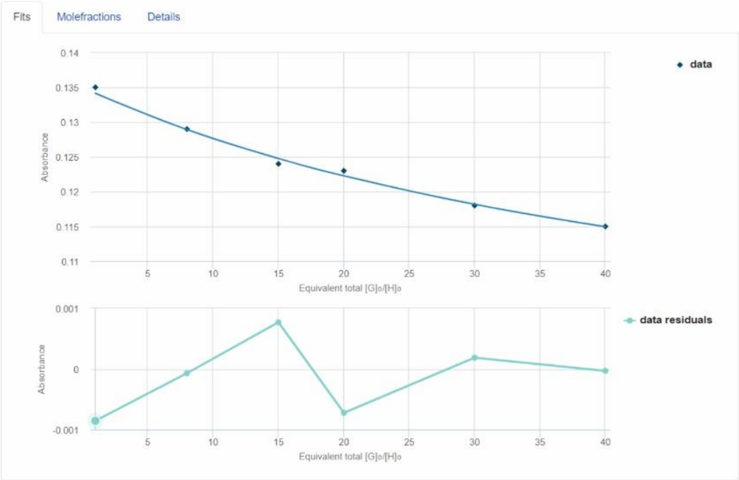

(B)

Fitter: UV 1:2 | Fit | Summary | Save

Details

**Time to fit**	0.8484 s
**SSR**	1.2385e-6
**Fitted datapoints**	6
**Fitted params**	4

Parameters

Parameter (bounds)	Optimised	Error	Initial
K_{11} (0 → ∞)	152009581692571008.00 M^{-1}	± 1327040882.8891 %	1000. M^{-1}
K_{12} (0 → ∞)	2400.13 M^{-1}	± 25.2300 %	100.0 M^{-1}

Back | Next

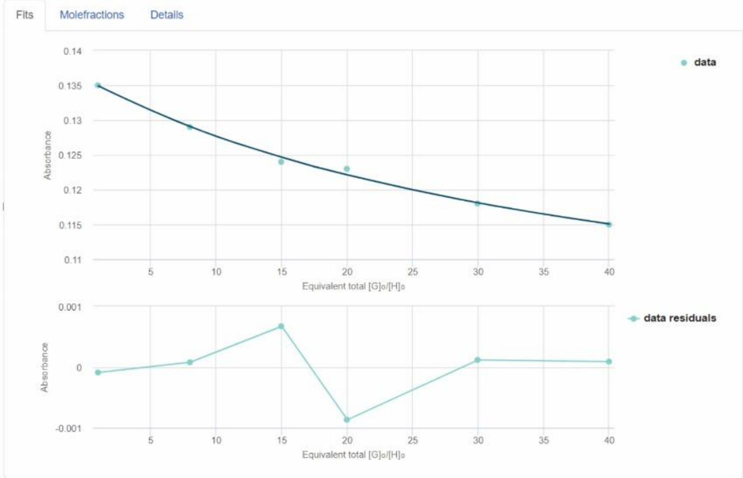

(C)

Fitter: UV 2:1 | Fit | Summary | Save

Details

**Time to fit**	0.8791 s
**SSR**	1.0864e-6
**Fitted datapoints**	6
**Fitted params**	4

Parameters

Parameter (bounds)	Optimised	Error	Initial
K_{11} (0 → ∞)	3662.85 M^{-1}	± 330.1660 %	1000.00 M^{-1}
K_{21} (0 → ∞)	2338451.31 M^{-1}	± 354.3563 %	100.00 M^{-1}

Back | Next

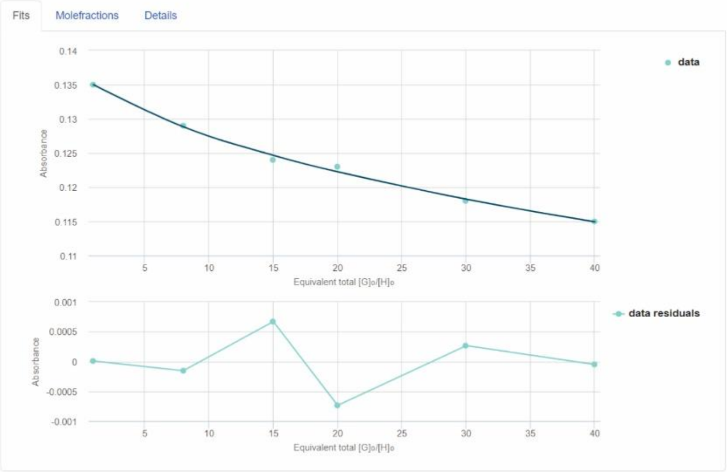

Figure S29. Screenshots taken from the summary window of the website supramolecular.org. This screenshots shows the raw data for UV-vis titration of 7 with Fluorescein, the data fitted to 1:1 binding model (A), 1:2 binding model (B) and 2:1 binding model (C).

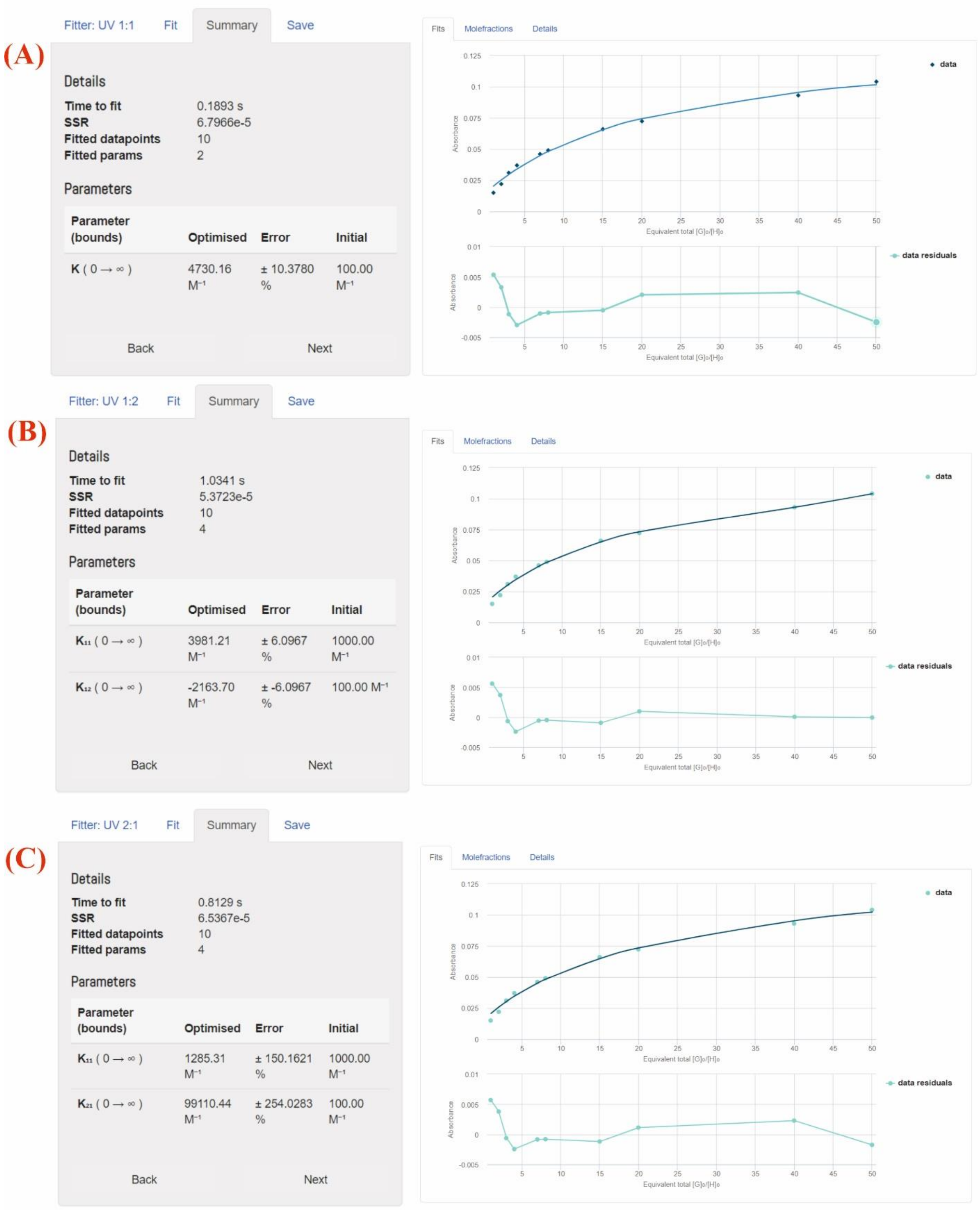

Figure S30. Screenshots taken from the summary window of the website supramolecular.org. This screenshots shows the raw data for UV-vis titration of 9 with Fluorescein, the data fitted to 1:1 binding model (A), 1:2 binding model (B) and 2:1 binding model (C).

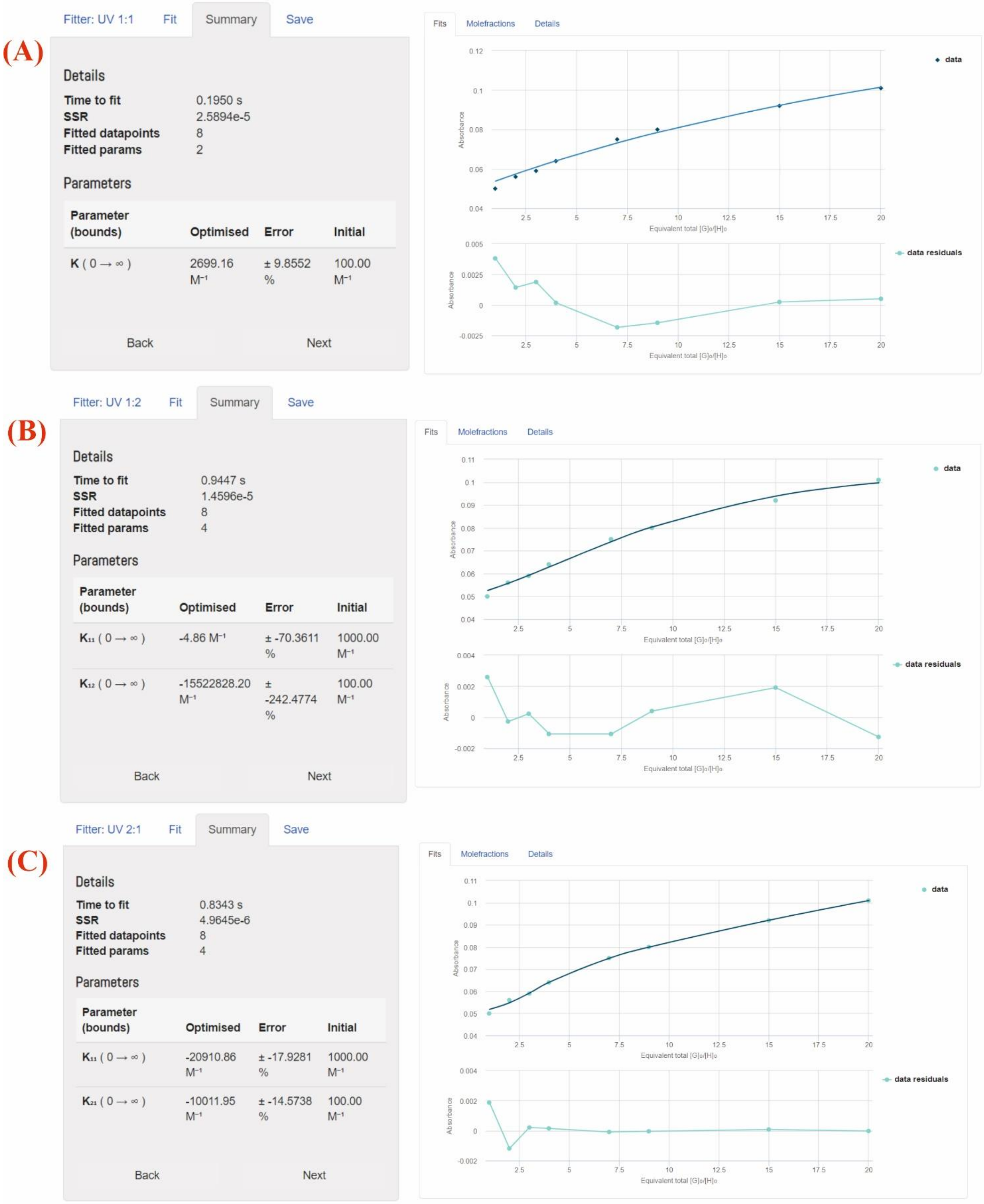

Figure S31. Screenshots taken from the summary window of the website supramolecular.org. This screenshots shows the raw data for fluorescence titration of 7 with Fluorescein, the data fitted to 1:1 binding model (A), 1:2 binding model (B) and 2:1 binding model (C).

(A)

Fitter: UV 1:1 | Fit | Summary | Save

Details

**Time to fit**	0.1942 s
**SSR**	13495131713.9540
**Fitted datapoints**	9
**Fitted params**	2

Parameters

Parameter (bounds)	Optimised	Error	Initial
K (0 → ∞)	10614.09 M^{-1}	± 10.0111 %	100.00 M^{-1}

Back Next

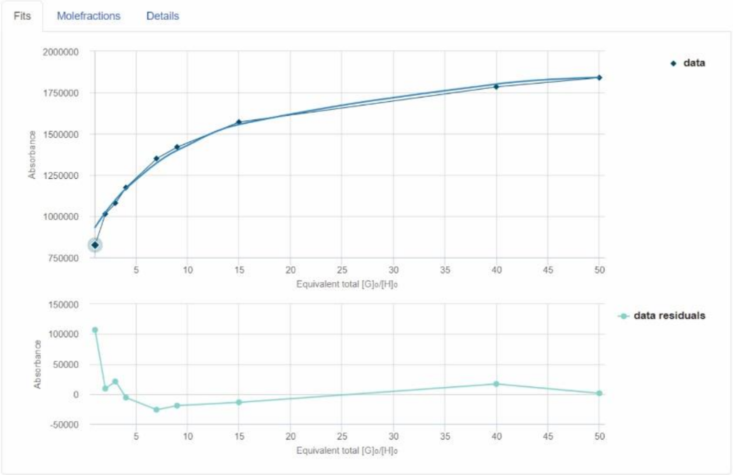

(B)

Fitter: UV 1:2 | Fit | Summary | Save

Details

**Time to fit**	0.9734 s
**SSR**	2642467558.3104
**Fitted datapoints**	9
**Fitted params**	4

Parameters

Parameter (bounds)	Optimised	Error	Initial
K_{11} (0 → ∞)	38234790356612352.00 M^{-1}	± 67084809.1027 %	1000.00 M^{-1}
K_{12} (0 → ∞)	14527.24 M^{-1}	± 11.7228 %	100.00 M^{-1}

Back Next

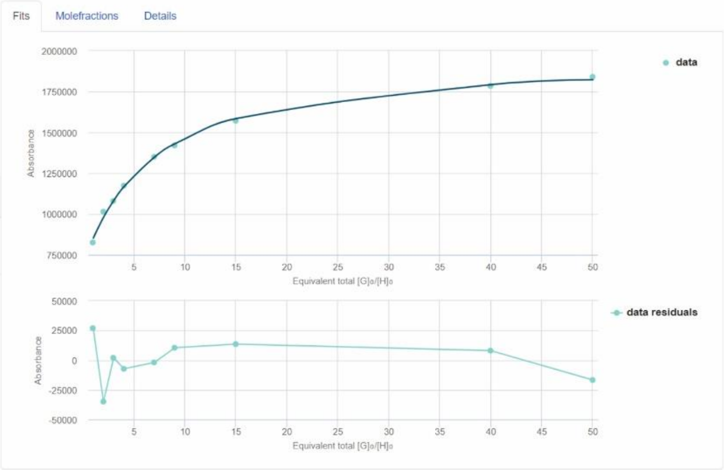

(C)

Fitter: UV 2:1 | Fit | Summary | Save

Details

**Time to fit**	0.9015 s
**SSR**	1186825413.6849
**Fitted datapoints**	9
**Fitted params**	4

Parameters

Parameter (bounds)	Optimised	Error	Initial
K_{11} (0 → ∞)	227365.07 M^{-1}	± 96.7122 %	1000.00 M^{-1}
K_{21} (0 → ∞)	1005266.52 M^{-1}	± 97.6923 %	100.00 M^{-1}

Back Next

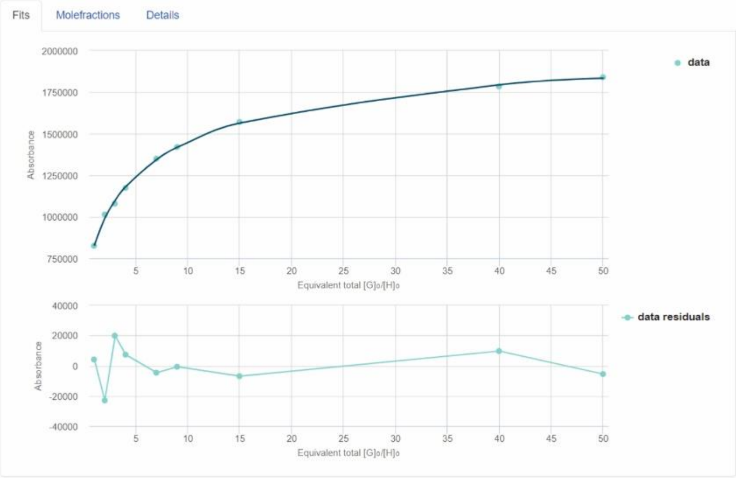

4. Table S3. Dynamic light scattering.

Aggregation of the particles for 7 / Flu and 7 / PVTE in EtOH.

Ratio 7 / Flu	V, µl	C_7, M	C_{Flu}, M	Z average (d) , nm	PDI	ζ- potential, mV
1:0	1000	10^{-3}	0	376.40±34.49	0.42±0.12	-
1:0	1000	10^{-4}	0	406.60±71.45	0.35±0. 10	-
1:0	1000	10^{-5}	0	760.20±111.20	0.44±0.29	-
1:1	1000	10^{-3}	10^{-3}	428.30±8.12	0.36±0.08	3.20±0.10
1:2	1000	10^{-3}	2×10^{-3}	433.90±102.40	0.41 ± 0.02	-
2:1	1000	2×10^{-3}	10^{-3}	305.70±48.18	0.43±0.01	-
1:1	1000	10^{-4}	10^{-4}	336.00±13.60	0.33±0.02	1.40±0.72
1:2	1000	10^{-4}	2×10^{-4}	457.50±149.50	0.47±0.13	-
2:1	1000	2×10^{-4}	10^{-4}	456.80±131.40	0.45±0.11	-
1:1	1000	10^{-5}	10^{-5}	155.40±7.16	0.16±0.02	5.94±0.06
1:2	1000	10^{-5}	2×10^{-5}	225.40±7.12	0.23±0.02	2.50±0.58
2:1	1000	2×10^{-5}	10^{-5}	191.00±25.87	0.26±0.05	2.74±0.14
0:1	1000	0	10^{-3}	460	1	-
0:1	1000	0	10^{-4}	-	-	-
0:1	1000	0	10^{-5}	2328	1	-
**7 / PVTE**	**V, µl**	**C_7, M**	**C_{PVTE}, M**	**Z average (d) , nm**	**PDI**	**ζ- potential, mV**
50:1	1000	5×10^{-4}	10^{-5}	213.22±4.11	0.31±0.07	-5.44±0.08
10:1	1000	10^{-4}	10^{-5}	116.01±2.26	0.23±0.01	-9.21±0.05
5:1	1000	5×10^{-5}	10^{-5}	198.44±8.10	0.34±0.15	-
2:1	1000	2×10^{-5}	10^{-5}	302.11±1.87	0.35±0.05	-
1:1	1000	10^{-5}	10^{-5}	417.20±11.18	0.39±0.11	-
1:2	1000	10^{-5}	2×10^{-5}	405.54±10.05	0.41±0.17	-
1:5	1000	10^{-5}	5×10^{-5}	440.56±18.16	0.46±0.15	-
1:15	1000	10^{-5}	1.5×10^{-4}	315.20±5.23	0.37±0.28	-
0:1	1000	0	10^{-3}	670.30±456.40	0.60±0.19	-
0:1	1000	0	10^{-4}	108.80±21.02	0.41±0.09	-
0:1	1000	0	10^{-5}	678.00±486.90	0.52±0.17	-

Aggregation of the particles for 9 / Flu and 9 / PVTE in EtOH.

Ratio 9 / Flu	V, µl	C_9, M	C_{Flu}, M	Z average (d), nm	PDI	ζ- potential, mV
1:0	1000	10^{-3}	0	690.10±93.47	0.56±0.34	-
1:0	1000	10^{-4}	0	255.80±96.04	0.58±0. 24	-
1:0	1000	10^{-5}	0	262.30±166.60	0.45±0.14	-
1:1	1000	10^{-3}	10^{-3}	733.70±209.60	0.48±0.37	-
1:2	1000	10^{-3}	2×10^{-3}	1448.00±72.40	0.47 ± 0.12	-
2:1	1000	2×10^{-3}	10^{-3}	760.80±20.55	0.49±0.13	-
1:1	1000	10^{-4}	10^{-4}	876.40±438.80	0.62±0.16	-
1:2	1000	10^{-4}	2×10^{-4}	451.10±221.10	0.44±0.21	-
2:1	1000	2×10^{-4}	10^{-4}	363.20±167.30	0.40±0.14	-
1:1	1000	10^{-5}	10^{-5}	273.50±162.90	0.31±0.16	0.05±0.03
1:2	1000	10^{-5}	2×10^{-5}	309.90±37.24	0.40±0.12	-
2:1	1000	2×10^{-5}	10^{-5}	408.80±176.60	0.45±0.14	-

9 / PVTE	V, μl	C_9, M	C_{PVTE}, M	Z average (d), nm	PDI	ζ- potential, mV
50:1	1000	5×10^{-4}	10^{-5}	312.25±7.12	0.31±0.07	-
10:1	1000	10^{-4}	10^{-5}	125.10±4.15	0.40±0.09	-
5:1	1000	5×10^{-5}	10^{-5}	270.21±5.14	0.38±0.23	-
2:1	1000	2×10^{-5}	10^{-5}	288.60±3.13	0.39±0.10	-
1:1	1000	10^{-5}	10^{-5}	339.45±10.21	0.40±0.08	-
1:2	1000	10^{-5}	2×10^{-5}	550.28±24.35	0.48±0.21	-
1:5	1000	10^{-5}	5×10^{-5}	1120.50±56.40	0.50±0.21	-
1:15	1000	10^{-5}	1.5×10^{-4}	-	-	-

Aggregation of the particles for 7/Flu/PVTE in EtOH.

7/Flu/PVTE	V, μl	C_7, M	C_{Flu}, M	C_{PVTE}, M	Z average (d), nm	PDI	ζ- potential, mV
1:1:0.1	1000	10^{-3}	10^{-3}	10^{-4}	418.24±10.11	0.41±0.12	-
1:1:1	1000	10^{-3}	10^{-3}	10^{-3}	312.15±5.10	0.35±0.14	-
1:1:5	1000	10^{-3}	10^{-3}	5×10^{-3}	214.74±10.08	0.33±0.04	-6.13±0.11
1:1:10	1000	10^{-3}	10^{-3}	10^{-2}	54.11±1.12	0.20±0.04	-11.15±0.10
1:1:0.1	1000	10^{-4}	10^{-4}	10^{-5}	502.11±20.43	0.54±0.21	-
1:1:1	1000	10^{-4}	10^{-4}	10^{-4}	395.00±66.37	0.36±0.04	-8.25±0.54
1:1:5	1000	10^{-4}	10^{-4}	5×10^{-4}	188.23±9.17	0.38±0.09	-9.16±0.36
1:1:10	1000	10^{-4}	10^{-4}	10^{-3}	51.04±2.07	0.21±0.04	-10.79±0.08
1:1:0.1	1000	10^{-5}	10^{-5}	10^{-6}	287.20±34.17	0.32±0.07	-7.14±0.24
1:1:1	1000	10^{-5}	10^{-5}	10^{-5}	114.30±2.10	0.20±0.01	-10.45±0.07
1:1:5	1000	10^{-5}	10^{-5}	5×10^{-3}	102.20±2.24	0.21±0.05	-11.04±0.17
1:1:10	1000	10^{-5}	10^{-5}	10^{-4}	48.02±1.10	0.16±0.01	-12.81±0.04

Aggregation of the particles for 7/Flu/PVTE in H_2O/EtOH (100/1).

7/Flu/PVTE	V_{H2O}, μl	V_{EtOH}, μl	C_7, M	C_{Flu}, M	C_{PVTE}, M	Z average (d), nm	PDI	ζ- potential, mV
1:1:0.1	1000	10	10^{-3}	10^{-3}	10^{-4}	388.58±9.23	0.64±0.25	-
1:1:1	1000	10	10^{-3}	10^{-3}	10^{-3}	324.90±7.80	0.53±0.02	-
1:1:5	1000	10	10^{-3}	10^{-3}	5×10^{-3}	183.45±5.15	0.36±0.09	-9.23±1.47
1:1:10	1000	10	10^{-3}	10^{-3}	10^{-2}	68.26±1.12	0.09±0.01	-34.12±1.94
1:1:0.1	1000	10	10^{-4}	10^{-4}	10^{-5}	1560.40±145.24	1	-
1:1:1	1000	10	10^{-4}	10^{-4}	10^{-4}	417.40±54.39	0.42±0.15	-
1:1:5	1000	10	10^{-4}	10^{-4}	5×10^{-4}	245.36±2.23	0.35±0.04	-9.11±2.13
1:1:10	1000	10	10^{-4}	10^{-4}	10^{-3}	175.00±3.05	0.29±0.01	-26.40±1.45

Aggregation of the particles for 7/Flu/PVTE in buffer (pH 9-2).

7/Flu/PVTE	V buff., µl (pH)	V EtOH, µl	C_7 (EtOH), M	C_{Flu} (EtOH), M	C_{PVTE} (EtOH), M	Z average (d), nm	PDI
1:1:10	1000 (9)	10	10^{-3}	10^{-3}	10^{-2}	82.99±0.47	0.15±0.02
	1000 (7)	10	10^{-3}	10^{-3}	10^{-2}	84.33±0.72	0.12±0.01
	1000 (5)	10	10^{-3}	10^{-3}	10^{-2}	489.33±10.30	0.43±0.15
	1000 (4)	10	10^{-3}	10^{-3}	10^{-2}	688.40±25.69	0.63±0.09
	1000 (2)	10	10^{-3}	10^{-3}	10^{-2}	737.80±45.58	0.55±0.07

Figure S32. Size distribution of the particles by intensity for PVTE (1×10^{-5}M) in ethanol.

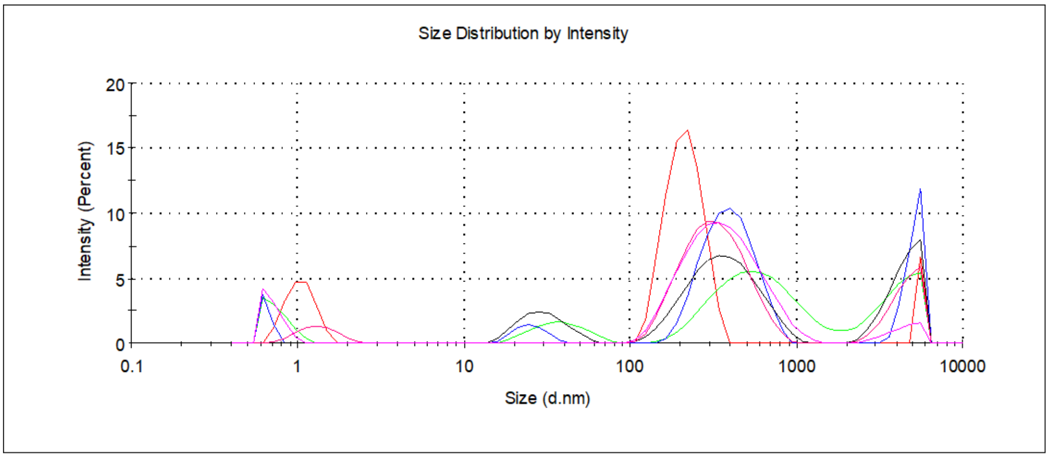

Figure S33. Size distribution of the particles by intensity for 7 (1×10^{-5}M) in ethanol.

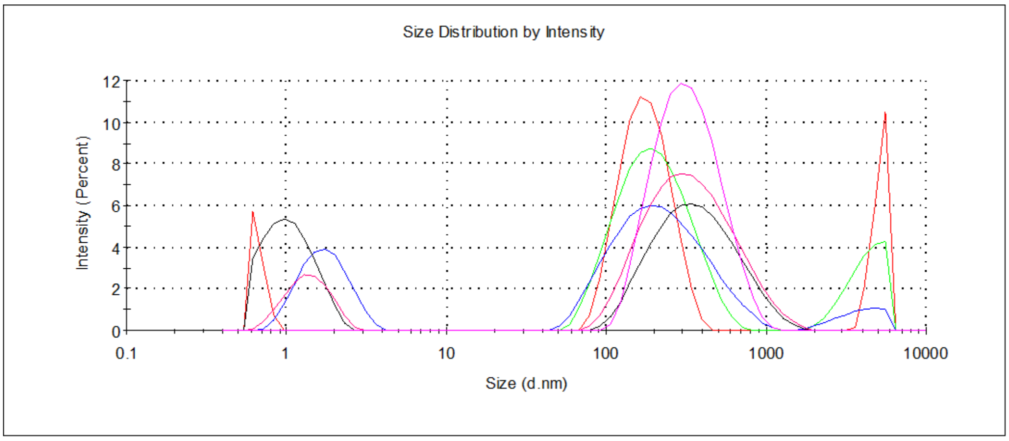

Figure S34. Size distribution of the particles by intensity for 7 (1×10^{-4} M) / PVTE (1×10^{-5} M) (10:1) in ethanol.

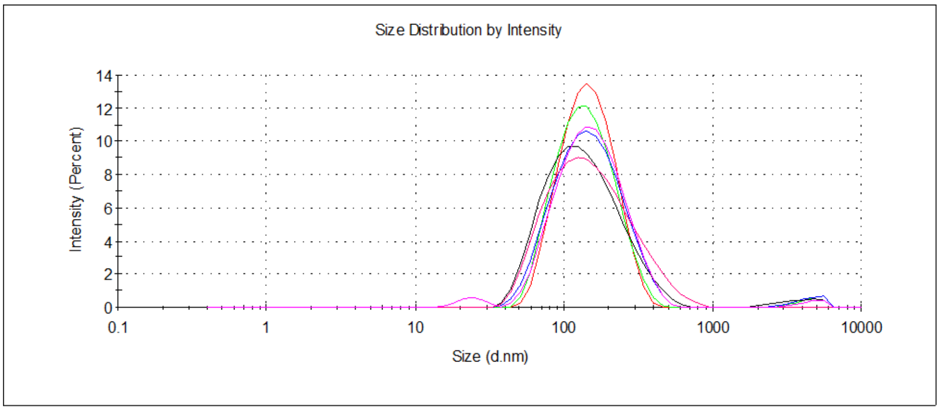

Figure S35. Size distribution of the particles by intensity for **7**/Flu, **7**/Flu/PVTE in ethanol and buffer at different pH.

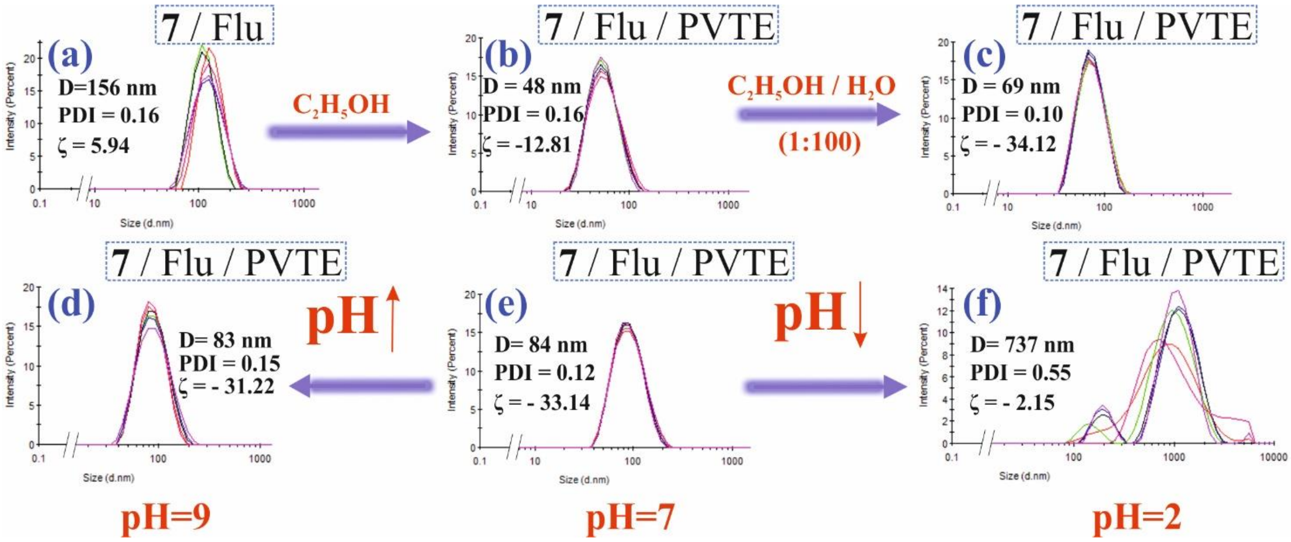

5. Scanning electron microscopy.

Figure S36. SEM image of silicon substrate.

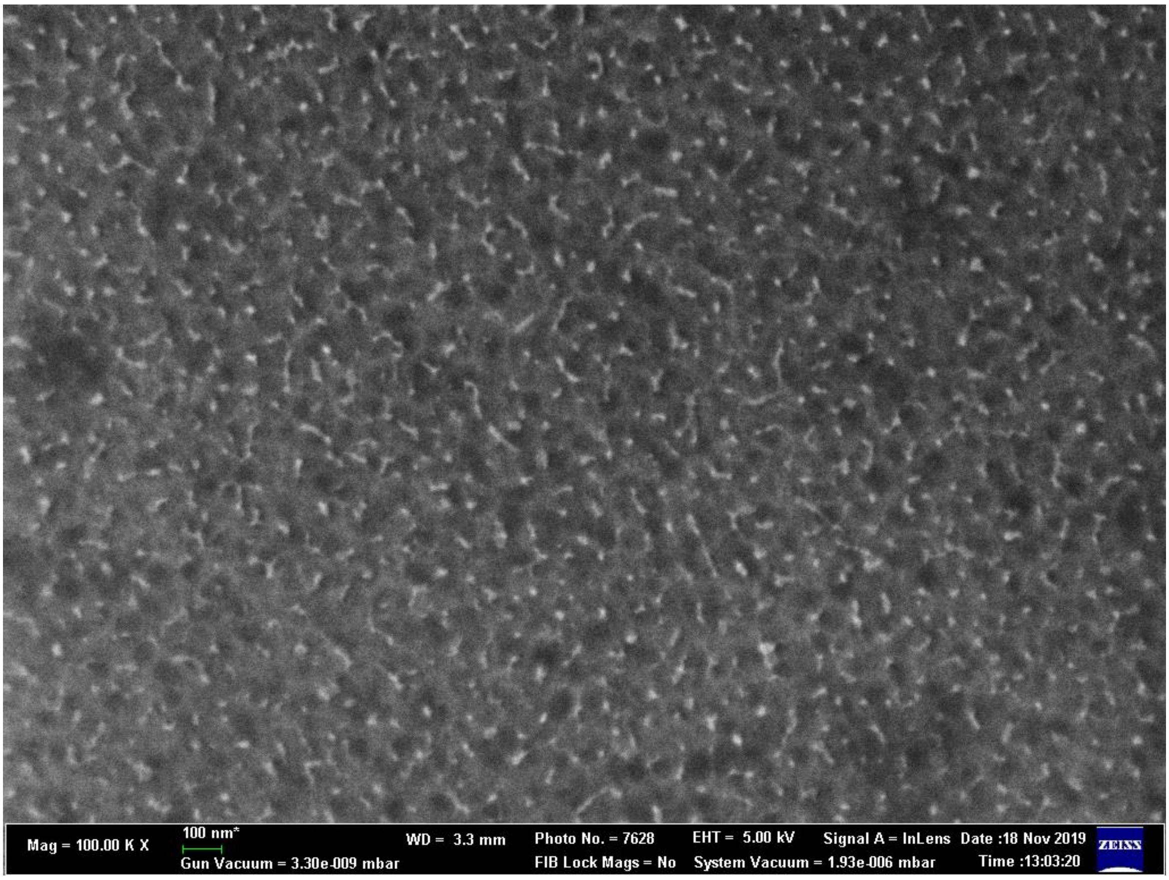

Figure S37. SEM image of 7/Flu/PVTE (1×10^{-5} M) after the solvent (H_2O/EtOH) evaporation.

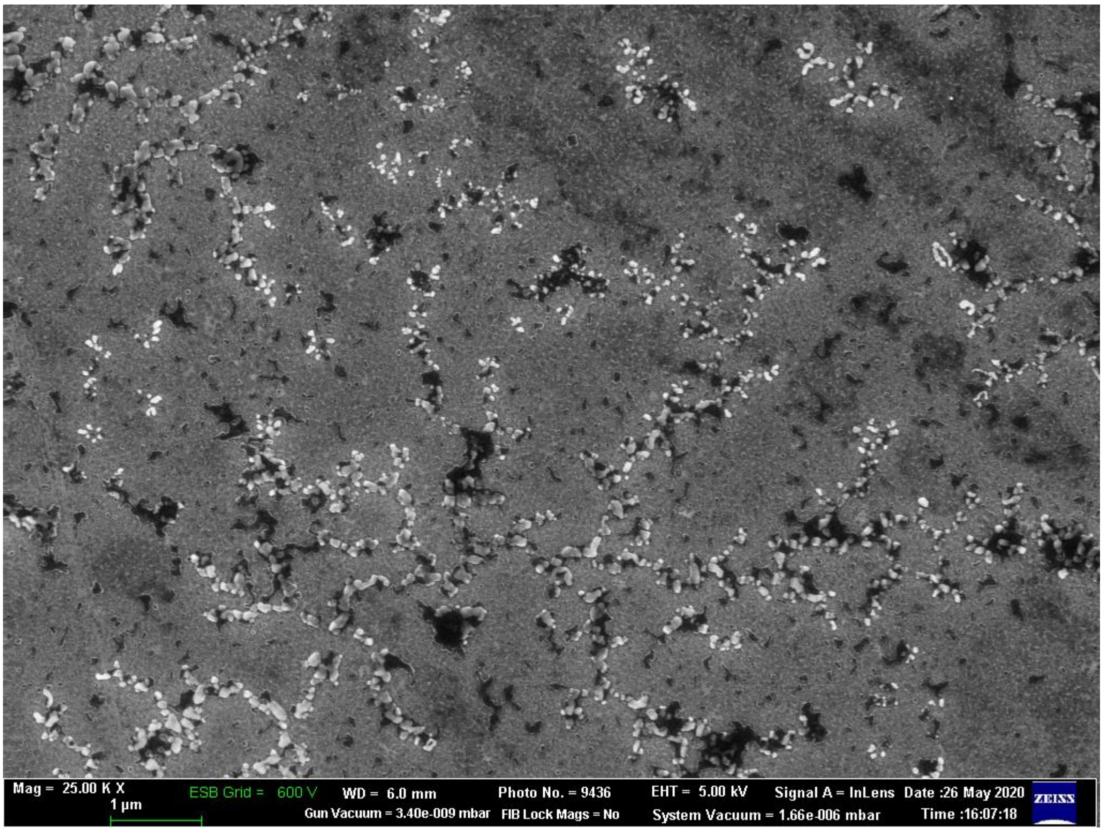

Figure S38. SEM image of 7/Flu/PVTE (1×10^{-5} M) after the solvent (H_2O/EtOH) evaporation.

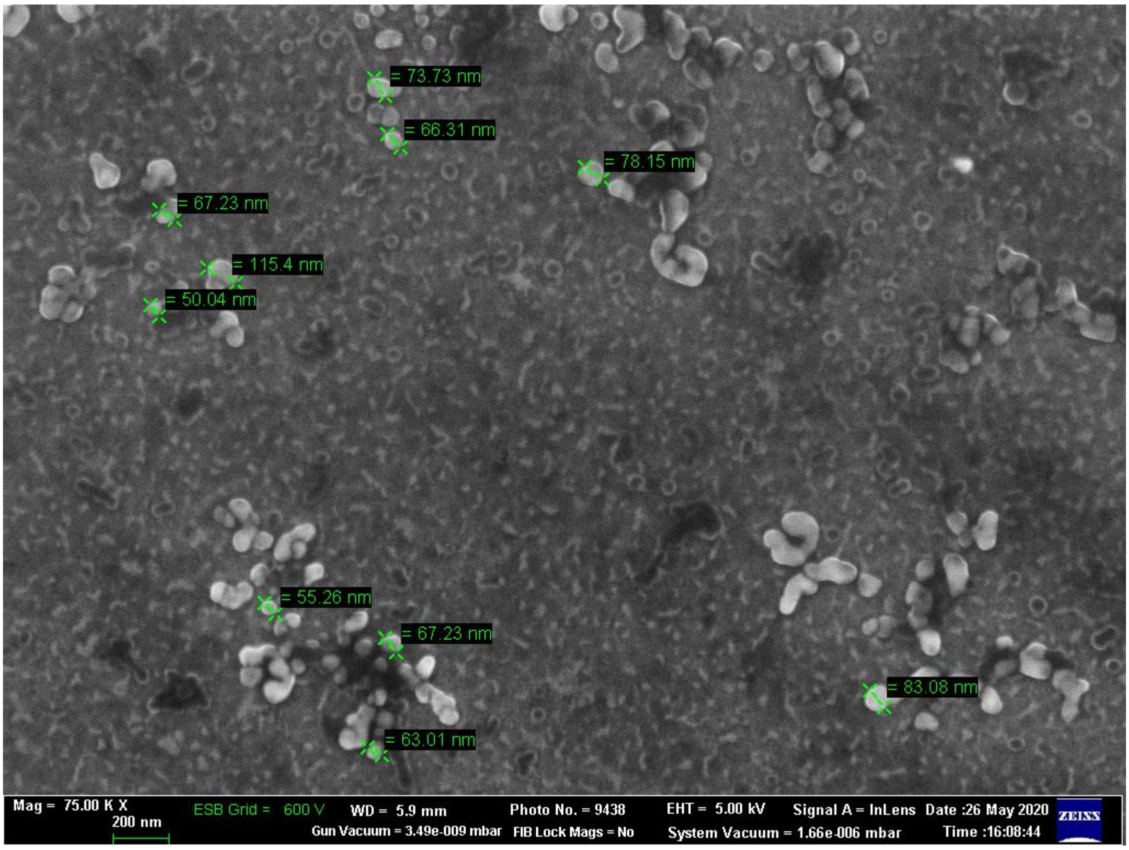

Figure S39. SEM image of 7/Flu/PVTE (1×10^{-5} M) after the solvent (H_2O/EtOH) evaporation.

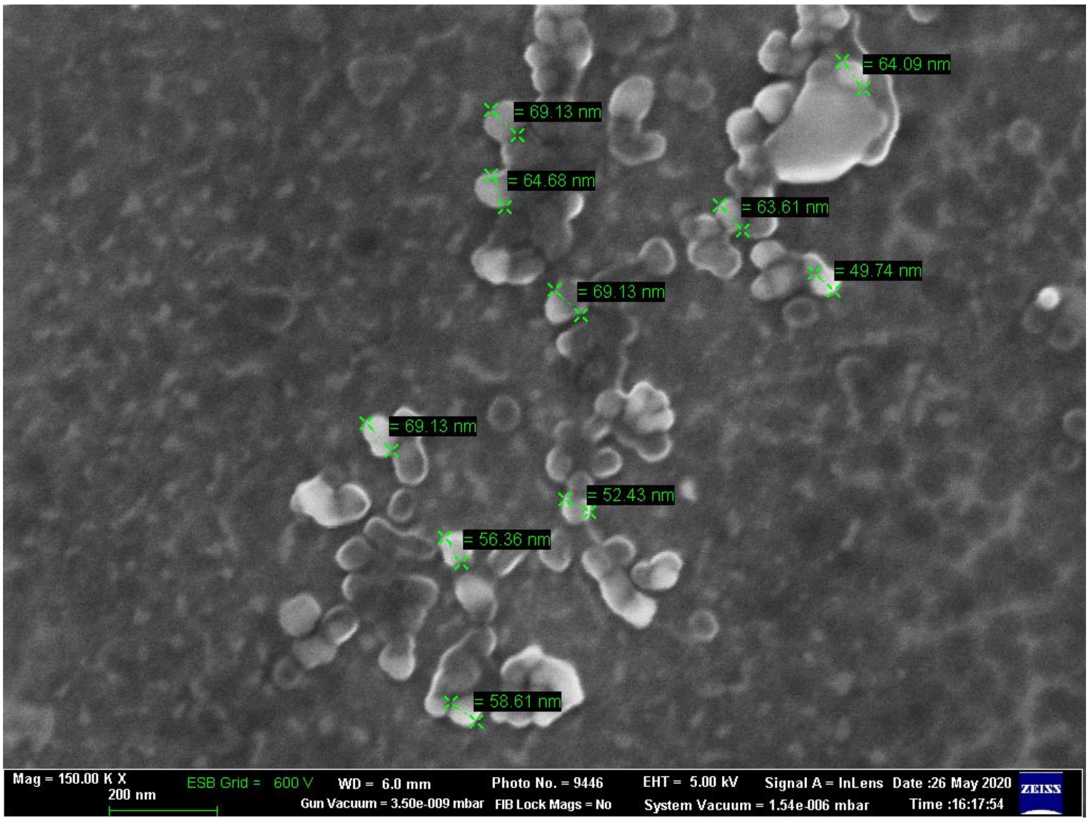

Figure S40. SEM image of 7/Flu (1×10^{-5} M) after the solvent (H_2O/EtOH) evaporation.

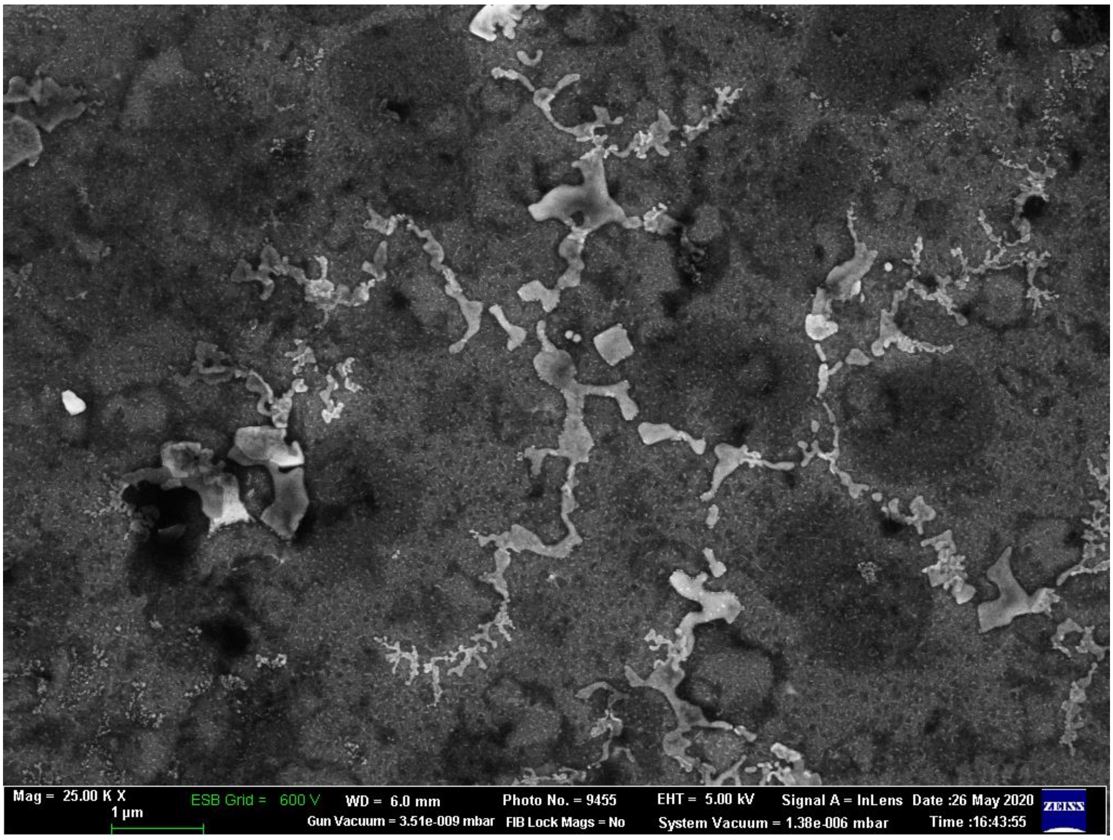

Figure S41. SEM image of PVTE (1×10^{-5} M) after the solvent (H_2O/EtOH) evaporation.

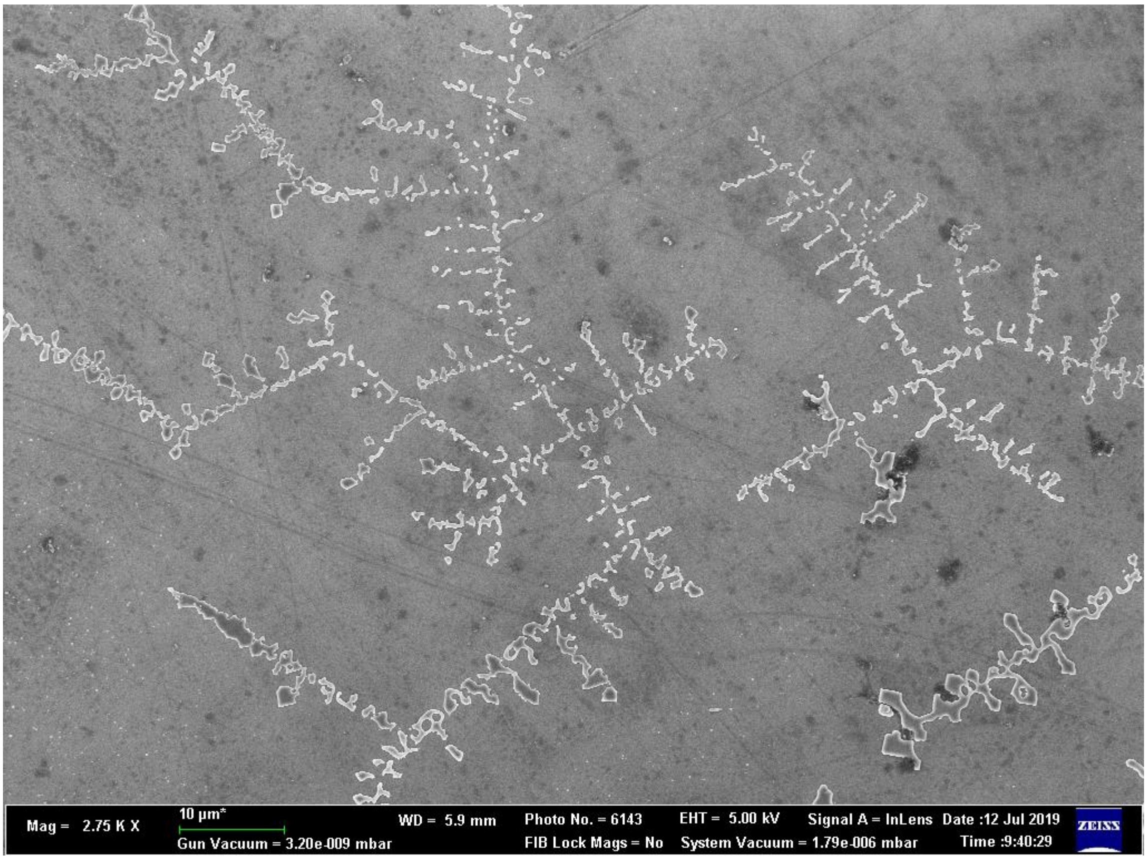

6. Fluorescence spectra

Figure S42. Fluorescence spectra of 7/Flu/PVTE (1×10^{-5}M) in buffer at different pH (9-2).

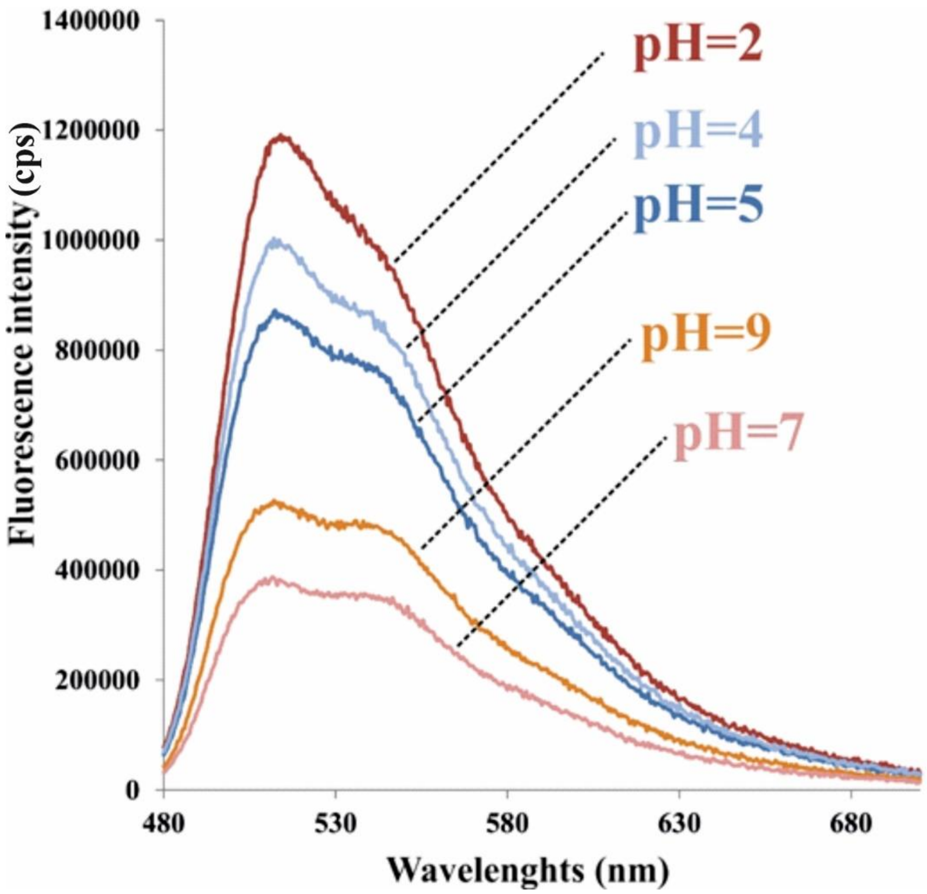

Supplement: Supplementary file 1 [file nanomaterials-11-00947-s001.pdf]
